# Profiling 26,000 *Aplysia californica* neurons by single cell mass spectrometry reveals neuronal populations with distinct neuropeptide profiles

**DOI:** 10.1016/j.jbc.2022.102254

**Published:** 2022-07-11

**Authors:** Peter C. Chan-Andersen, Elena V. Romanova, Stanislav S. Rubakhin, Jonathan V. Sweedler

**Affiliations:** Department of Chemistry and the Beckman Institute for Advanced Science and Technology, University of Illinois at Urbana-Champaign, Urbana, Illinois, USA

**Keywords:** mass spectrometry, neuropeptide, peptides, neuron, central nervous system, single cell, *Aplysia californica*, Louvain–Jaccard, apUII, *Aplysia* urotensin II, ARC, accessory radula closer, ASW, artificial sea water, BCN, bag cell neuron, BCP, bag cell peptide, CNS, central nervous system, ELH, egg laying hormone, ESI, electrospray ionization, ICC, immunocytochemistry, IHC, immunohistochemistry, ISH, *in situ* hybridization, KNN, k-nearest neighbors, LJ, Louvain–Jaccard, MS/MS, tandem mass spectrometry, MS, mass spectrometry, NPY, neuropeptide Y, PTM, posttranslational modification, ROI, region of interest, SCP, small cardiac peptide, S/N, signal-to-noise ratio

## Abstract

Neuropeptides are a chemically diverse class of cell-to-cell signaling molecules that are widely expressed throughout the central nervous system, often in a cell-specific manner. While cell-to-cell differences in neuropeptides is expected, it is often unclear how exactly neuropeptide expression varies among neurons. Here we created a microscopy-guided, high-throughput single cell matrix-assisted laser desorption/ionization mass spectrometry approach to investigate the neuropeptide heterogeneity of individual neurons in the central nervous system of the neurobiological model *Aplysia californica*, the California sea hare. In all, we analyzed more than 26,000 neurons from 18 animals and assigned 866 peptides from 66 prohormones by mass matching against an *in silico* peptide library generated from known *Aplysia* prohormones retrieved from the UniProt database. Louvain–Jaccard (LJ) clustering of mass spectra from individual neurons revealed 40 unique neuronal populations, or LJ clusters, each with a distinct neuropeptide profile. Prohormones and their related peptides were generally found in single cells from ganglia consistent with the prohormones’ previously known ganglion localizations. Several LJ clusters also revealed the cellular colocalization of behaviorally related prohormones, such as an LJ cluster exhibiting achatin and neuropeptide Y, which are involved in feeding, and another cluster characterized by urotensin II, small cardiac peptide, sensorin A, and FRFa, which have shown activity in the feeding network or are present in the feeding musculature. This mass spectrometry–based approach enables the robust categorization of large cell populations based on single cell neuropeptide content and is readily adaptable to the study of a range of animals and tissue types.

Neuropeptides are an important group of cell-to-cell signaling molecules formed by the posttranslational enzymatic cleavage of prohormones into bioactive peptides. Packaged in large dense core vesicles, neuropeptides often undergo further posttranslational modifications (PTMs), which are often required for bioactivity (1, 2). Once secreted, these signaling peptides are involved in the formation and modulation of an array of homoeostatic functions and behaviors, including mating, feeding, olfaction, pain, circadian rhythm, and addiction (3, 4, 5, 6, 7). Many neuropeptides appear to be multifunctional. Their simultaneous or differential release with other signaling molecules act on different cellular targets and often activate a variety of receptors. The chemical and functional heterogeneity of neuropeptides also include different secondary messenger pathways and variability in neuropeptide PTMs, which are critical for neural plasticity (2, 8). The diversity in neuropeptide expression, colocalization, and function can be observed even in neighboring cells; thus, having knowledge of an individual cell’s peptide content is critical for understanding normal and pathological neurobiology.

Peptide characterization has evolved from targeted studies to mass spectrometry (MS)-based peptidomic measurements, where global characterization of the peptides in a sample is possible. However, peptidomics relies on sample sizes that involve microliters of homogenized tissues (9, 10, 11, 12, 13, 14, 15, 16, 17, 18). While these studies characterize hundreds to thousands of peptides, they are unable to describe the underlying chemical heterogeneity on the single cell level and may miss rare but functionally important peptides that are present in only a few cells (19).

Single cell transcriptomics has been at the forefront of measuring and classifying large populations of individual cells (20). While revealing transcript colocalization, these approaches do not directly detect translated prohormones, processed mature peptides, or peptide PTMs. Alternatively, MS detects peptides, the final gene products, at single cell and subcellular levels (21, 22). Such single cell measurements are possible due to the low limits of detection, high dynamic range, untargeted detection, and broad analyte coverage provided by MS. Techniques such as electrospray ionization (ESI)-MS (23, 24, 25), desorption ESI (26, 27), capillary electrophoresis-ESI-MS (28, 29, 30, 31, 32), secondary ion MS (33, 34), and MALDI MS (19, 32, 35) have successfully characterized the chemical content of single cells.

We have created a MALDI MS workflow that, when applied to low density, dissociated single cells characterized by microscopy, acquires single cell mass spectra within several seconds (36), allowing thousands of cells to be profiled in a single experiment (37, 38, 39). Though these prior single cell MALDI MS studies focused on the characterization of lipids in tens of thousands of cells (38, 40) and produced robust data that can be used to classify cells (41), the approach has also been used to characterize single organelles (42) and individual cells from endocrine structures, including islets and the pituitary from rats (19, 43).

The overarching goal in this work was to create and demonstrate a global single cell workflow for peptides in order to characterize the neuropeptide complexity of the brain using the marine mollusk *Aplysia californica* as a model system. *Aplysia* is a well-characterized neurobiological organism that has been used for neuropeptide investigations for several decades (44). As compared with more complex mammalian models, the *Aplysia* central nervous system (CNS) contains only ∼20,000 neurons, with soma ranging in size from 5 to 1000 μm (45). Many *Aplysia* prohormones and neuropeptides have been identified using various molecular techniques (46, 47, 48, 49), transcriptomics (50), manual isolation of key individual cells and performing single cell MS (46, 51, 52, 53), and liquid chromatography-tandem mass spectrometry (MS/MS) (9, 54, 55). Known roles for many *Aplysia* neuropeptides include egg laying (56), cardiovascular activity (57), feeding (48, 51, 52, 58, 59, 60, 61, 62, 63), respiratory pumping (64), siphon-withdrawal (65), and glucose metabolism (66). The wealth of available neuropeptide information on the *Aplysia* CNS made it an ideal organism to demonstrate this high-throughput, single cell MS protocol. Another goal was to create a method for commonly available MALDI-TOF mass spectrometers so as to facilitate its implementation by other laboratories.

This work combined the anatomical advantages and neurochemical information of the *Aplysia* CNS with high-throughput single cell profiling using microscopy-guided MALDI MS to sample and classify 26,797 *Aplysia* single neurons. Mass spectral features were assigned to peptides predicted *in silico* from known *Aplysia* prohormones retrieved from UniProt (67). Statistical analysis using the Louvain–Jaccard (LJ) community detection algorithm (68) revealed 40 distinct LJ clusters, each defined by a unique single cell neuropeptide profile. This large-scale single cell cataloguing of neuropeptides within the *Aplysia* CNS reveals single cell chemical heterogeneity and peptide colocalization. These findings are expected to enable future studies of *Aplysia* neurobiology.

More broadly, we anticipate that this workflow will be well suited to working with the ever-increasing number of animal species for which we have genomic information but lack physiological or neuropeptide processing details. It provides an additional -omics approach (in addition to transcriptomics) (69, 70, 71) to differentiate populations of cells, in this case based on their important and functional neuropeptides and hormones.

## Results

### High-throughput peptide profiling of individual neurons

**Figure 1:**
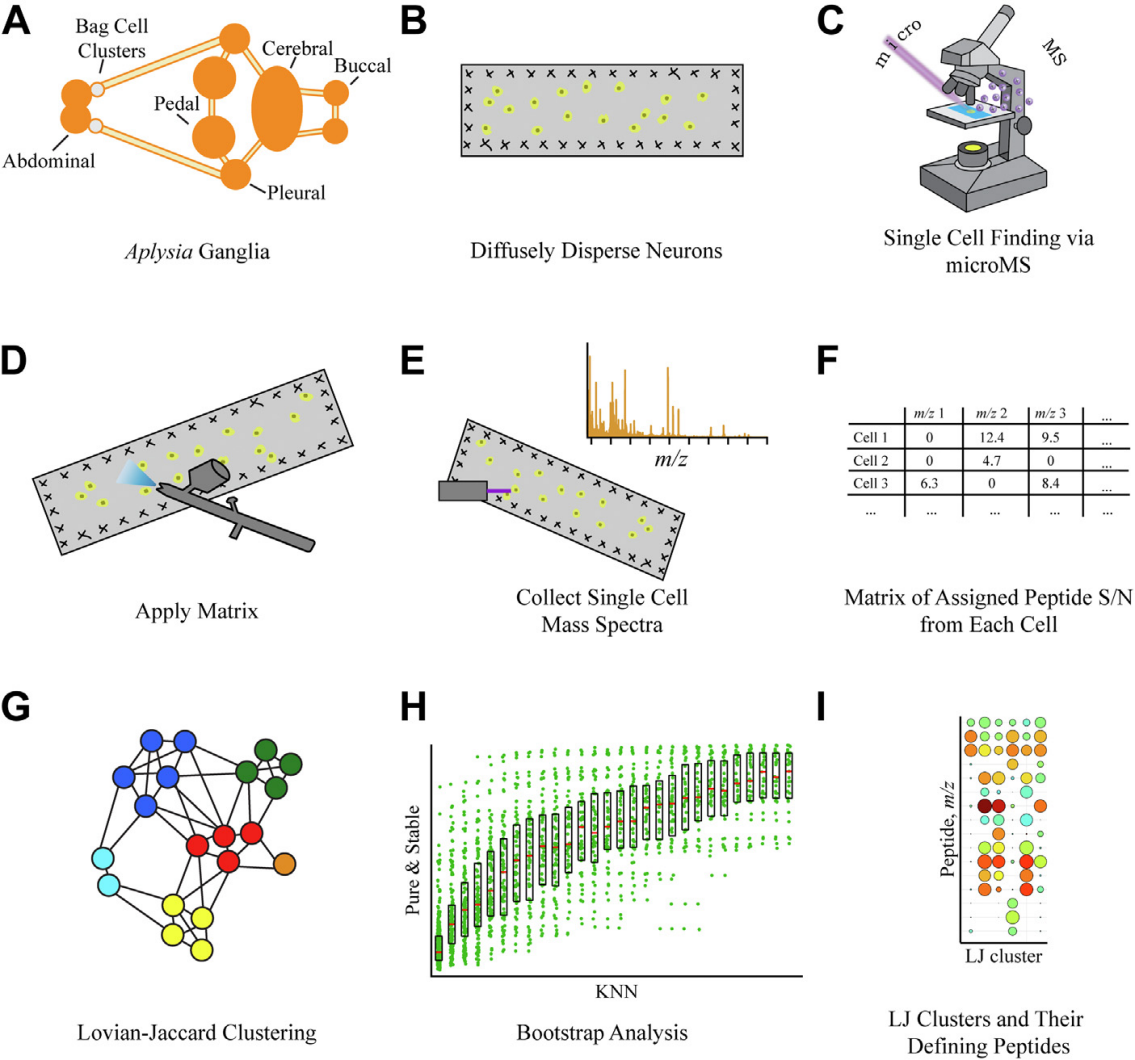
**Sample processing and data analysis workflow for high-throughput single cell mass spectrometry.***A*, *Aplysia* ganglia are dissected and individually isolated. *B*, stabilized ganglia are manipulated across slides to diffusely disperse individual cells. *C*, cells are identified and their positions recorded using microMS. *D*, MALDI matrix is applied. *E*, using the .xeo files from microMS single cells are sampled. *F*, peak lists are constructed for each cell by peptide mass fingerprinting using a 60-ppm mass error. *G*, the KNN of each cell is used to construct a KNN graph, which is Louvain–Jaccard (LJ) clustered. *H*, the appropriate KNN for LJ clustering is determined by bootstrap analysis. *I*, LJ clusters are defined by a unique colocalization of peptides. KNN, k-nearest neighbors; S/N, signal-to-noise ratio.

Neurons from each major *Aplysia* CNS ganglion (Table S1)—abdominal, bag cell clusters (bag cell neurons [BCNs]), buccal, cerebral, pleural, and pedal—were dispersed across indium tin oxide glass slides, fluorescently imaged, and sampled using MADLI MS guided by microMS (36). A 100-μm laser footprint was used to ensure only single neurons were sampled; cells located within 200 μm of one another were excluded, as well as objects that appeared to be nonneuronal (sheath, glia, debris, lysed cells) based on their shape and/or size. The collected mass spectra were peak picked for mass spectral signals with a signal-to-noise ratio (S/N) greater than five (noise was calculated as the standard deviation of intensities below the 99th percentile in a 200 *m/z* window around each point). Each cell’s peak list was matched to a database of *in silico* predicted *Aplysia* peptides. To assign a peptide from a given prohormone, at least two peptides from that prohormone must be assigned in the same peak list, unless the prohormone produced three or fewer peptides. Owing to intense lipid signals in the 600 to 900 mass-to-charge ratio (*m/z*) range, only *m/*z greater than 900 were investigated (Fig. 1).

Using this approach, 26,797 cells were sampled that contained mass spectrometric features mass matched to an *in silico* peptide library in the 900- to 5500-*m/z* range. Sampled cells ranged from 10 to 300 μm, with an average size of 67 ± 34 μm (mean ± standard deviation) and a median size of 59 μm (Fig. S1). Our cell isolation approach limits our ability to isolate and characterize *Aplysia* neurons larger than 300 μm, and so these do not appear in this study. A total of 866 peptides from 66 prohormones were assigned. This corresponds to 65% of the 1333 peptides and 92% of the 72 prohormones in the *Aplysia in silico* library. Of the peptides that were not assigned, 324 had a monoisotopic *m/z* below 900. This represents 24% of all the peptides in the library, meaning that 866 peptides were assigned from the remaining 1009 peptides (86% assigned of peptides >900 *m/z*). A primary limitation in assigning single cell MALDI mass spectral features is the difficulty in acquiring MS/MS measurements. The low amount of peptide in an individual neuron and the generally poor fragmentation of singly charged ions result in uninformative MS/MS results. Therefore, a peptide mass fingerprinting approach was used that required two or more peptides from the same prohormone to be matched with a mass error <60 ppm before assignment. Unknown features and isobaric peptides with the 60-ppm mass error could not be assigned, which may explain several hundred detected but unassigned spectral features in the data set. The identification of these features will be the subject of future studies.

### Peptide profile–based LJ clustering of large neuronal populations

**Figure 2:**
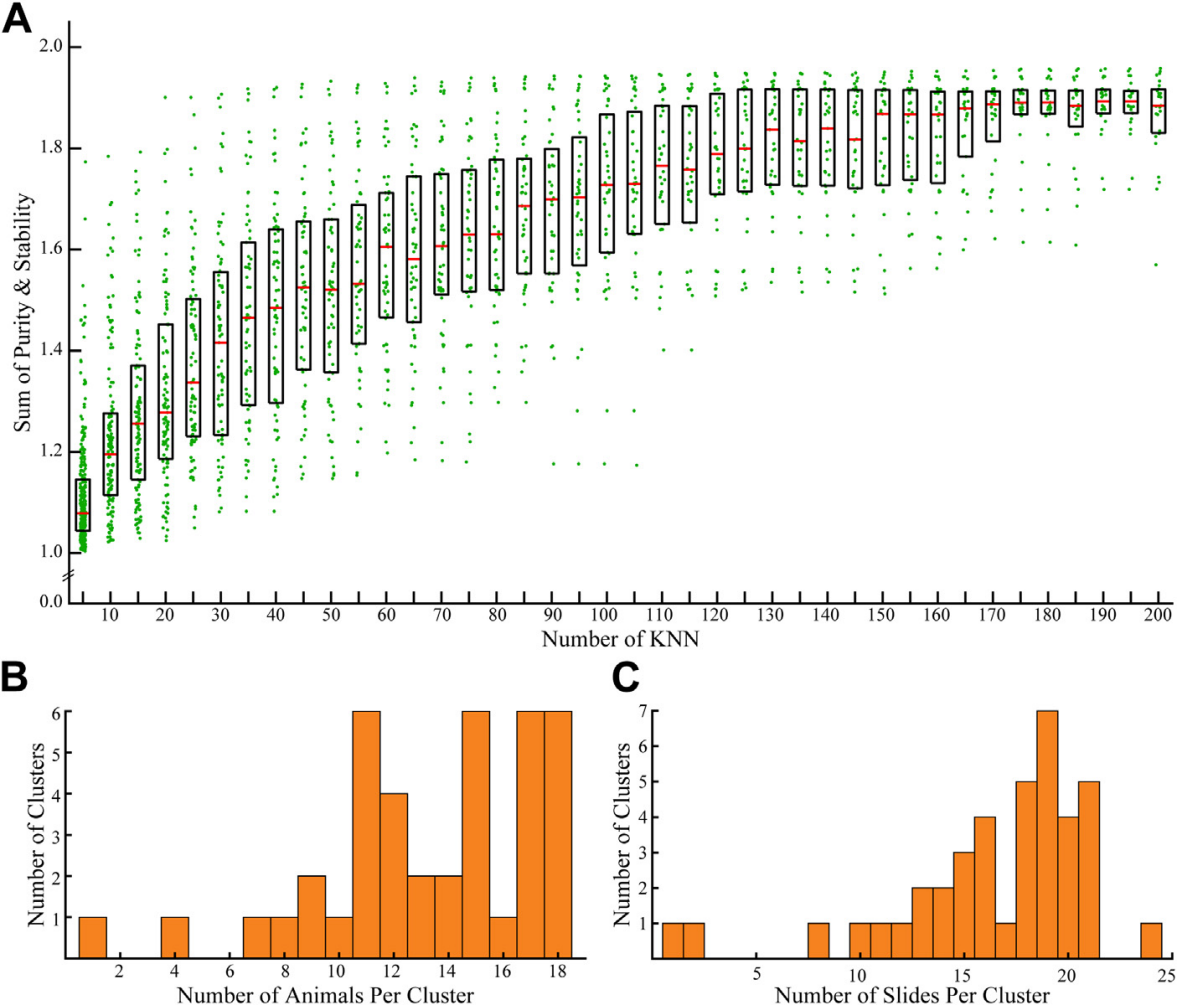
**Results of LJ clustering and bootstrapping analysis of data obtained using high-throughput single cell mass spectrometry peptide profiling.***A*, results of the bootstrapping experiment. Each dot represents the sum of stability and purity metrics for each all-cell set LJ cluster at a given KNN. The *black boxes* denote the range between the 75th and 25th percentiles, and the red bars mark the median value. The plateauing of stability and purity after KNN 100 indicates it as the optimal KNN for the data set. *B*, histogram showing the number of animals represented in each of the 40 clusters resulting from 100 KNN LJ clustering after merging redundant clusters; a total of 18 animals were used. An animal was determined to contribute to a cluster if more than 2% of a cluster’s cell came from a given animal. *C*, histogram showing the number of indium tin oxide glass slides whose cells contributed to each of the 40 clusters resulting from 100 KNN LJ clustering after merging redundant clusters. In total, 52 sample slides were used. A slide was determined to contribute to a cluster if more than 2% of a cluster’s cells came from a given slide. KNN, k-nearest neighbors; LJ, Louvain–Jaccard.

To reveal and characterize cellular phenotypes, the *Aplysia* neuronal profiles were grouped using LJ clustering, which requires selecting an appropriate number of k-nearest neighbors (KNNs). KNN determines the *k* number of most similar cells for each of the 26,797 cells (where *k* is a positive integer). Each neighboring pair of cells is assigned an edge weight equal to their peptide Jaccard index. This network of KNN cells and their edge weights is used to construct a KNN graph on which the LJ clustering is run. In this case, the appropriate KNN is not known prior to analysis and so determining this value required bootstrapping. The 40 different all-cell LJ clustering outcomes (KNN 5–200, in steps of 5) were compared with 1000 bootstrapped LJ clustering outcomes (random KNN between 5 and 200). As the number of KNN increases in the all-cell sets, the stability and purity of the resultant LJ clusters also increases (Fig. 2*A*). As expected, this increase in stability and purity is accompanied by a decrease in the number of LJ clusters (i.e., a poorer description of the cellular heterogeneity). The optimal number of KNN is the point at which increasing the KNN no longer provides greater stability and purity while also diminishing the number of LJ clusters found.

In this case, KNN 100 appears as a potential candidate for the optimal KNN. The 105, 110, and 115 KNN outcomes have a similar range, 50th and 75th percentile values of purity and stability, and are located at the end of a roughly linearly increasing region of purity and stability. KNN 100 also includes a leveling in the number of LJ clusters produced as it had 41 clusters, whereas KNN 105, 110, and 115 produced 40, 38, and 38 clusters, respectively. Using a chi-square test of independence, comparing the cellular frequency of assigned peptides in each LJ cluster, KNN 100, 105, 110, and 115 contained the same two redundant LJ clusters. Redundant LJ clusters are those that differ not in peptide profile but in the S/N of the same peptide. For these reasons, KNN 100 was selected as the optimal number of KNNs.

The two redundant LJ clusters from KNN 100, determined by a chi-square test of independence, ultimately belonged to a single LJ cluster. The two redundant clusters only differed in the S/N of two peptides, monoisotopic *m/*z 1161.6 and 2770.5, from the sensorin A prohormone (P29233). Therefore, these two clusters were merged into the LJ cluster. The postmerge LJ clusters do not appear to be the result of animal-to-animal differences. The LJ clusters varied in size from several thousand cells in the largest LJ clusters to only around 100 in the smallest (Fig. S2). This suggests that a minimum ∼5 cells with the same peptide profile need be present in an animal in order for an LJ cluster to form. As a caveat to the approach, if there were an individual cell with a unique set of neuropeptides, it would be grouped with the LJ cluster with the most similar peptide content as singleton clustering was not allowed. Despite the variety of population sizes, 38 of the 40 LJ clusters contained cells collected from more than 7 of the 18 animals used in this study (Fig. 2*B*). The LJ clusters also comprised cells from many different slides prepared and sampled on different days (Fig. 2*C*). This indicates that variation in sample preparation quality or instrument performance did not substantially influence the clustering results. There were two anomalous clusters, the 190 cell LJ cluster 36 and the 129 cell LJ cluster 39. The cells in LJ clusters 36 and 39 came from four animals across two slides and one animal across one slide, respectively. Given their small cellular population, such LJ clusters are prone to come from relatively few animals and sampling slides.

**Figure 3:**
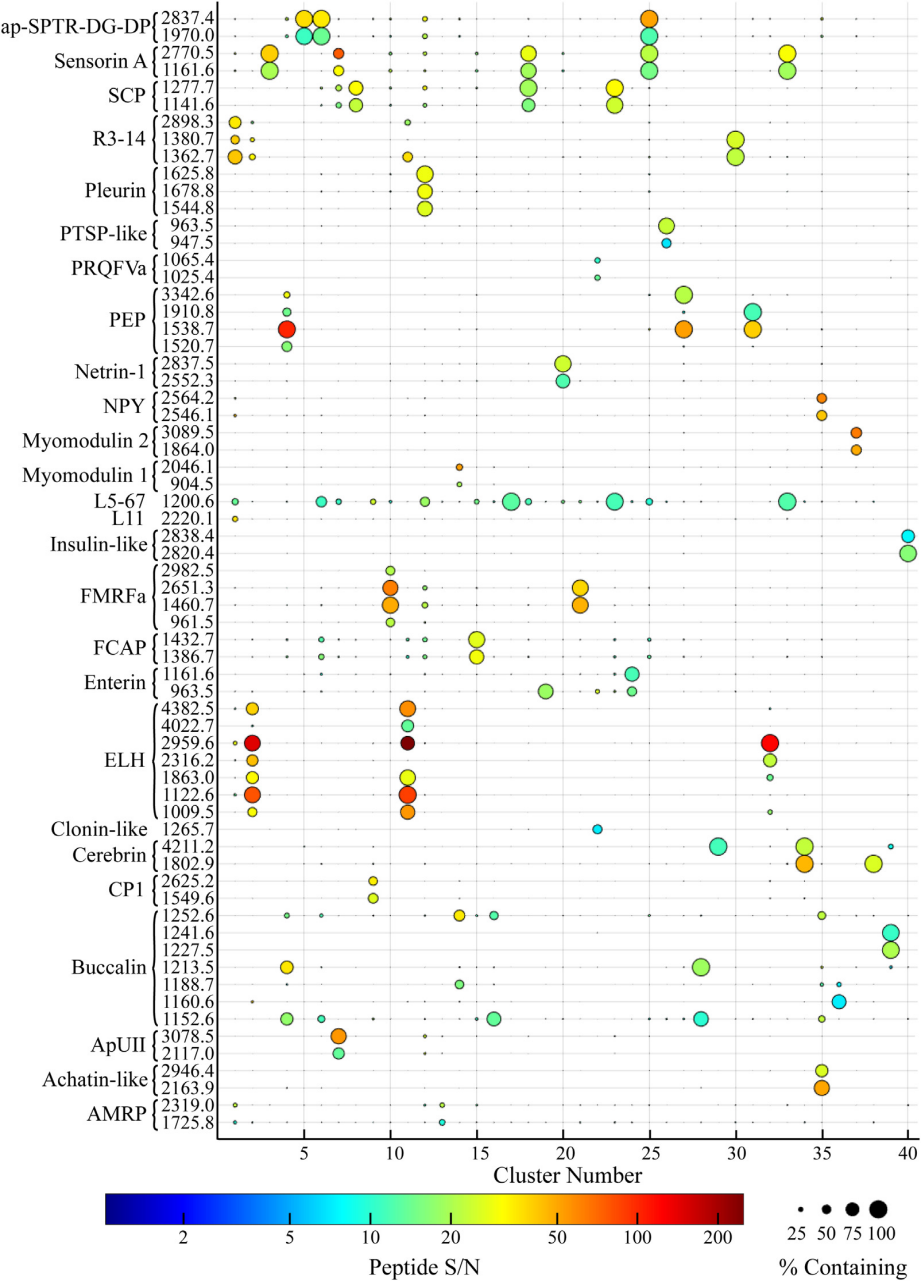
**LJ clustering of single *Aplysia californica* neuron mass spectra.** LJ clusters were determined using a k-nearest neighbors of 100 and the assigned peptides within each cell. As determined by a chi-square test of independence, a subset of 108 of the 866 assigned *Aplysia* peptides were found at different frequencies in at least one LJ cluster. Each cluster is defined by a unique combination of assigned peptides and/or cellular frequency of their detection. For clarity, only 65 peptides are shown (all 108 peptides can be seen in Fig. S3). The prohormone name and related peptide monoisotopic *m/z* is given on the ordinate. Circle size corresponds to a peptide’s frequency of detection in each cluster. Color scale depicts the average peptide signal-to-noise ratio (S/N) in a given cluster. LJ, Louvain–Jaccard.

After merging, 40 total LJ clusters were found in the analyzed neurons (Fig. 3), with each LJ cluster defined by a unique combination and/or cellular frequency of assigned peptides. The chi-square test of independence revealed that 108 of the 866 assigned peptides were found in a different cellular frequency in at least one of the 40 LJ clusters (Figs. 3 and S3).

### Single cell peptide mass fingerprinting analysis

For the majority of *Aplysia* prohormones, posttranslational processing results in the formation of more than one mature peptide, many of which have been shown to be bioactive (72, 73). Therefore, the detection of multiple peptides from the same prohormone in a single cell is expected and has been demonstrated in previous targeted studies of manually isolated single *Aplysia* neurons (52, 53, 74, 75, 76).

**Figure 4:**
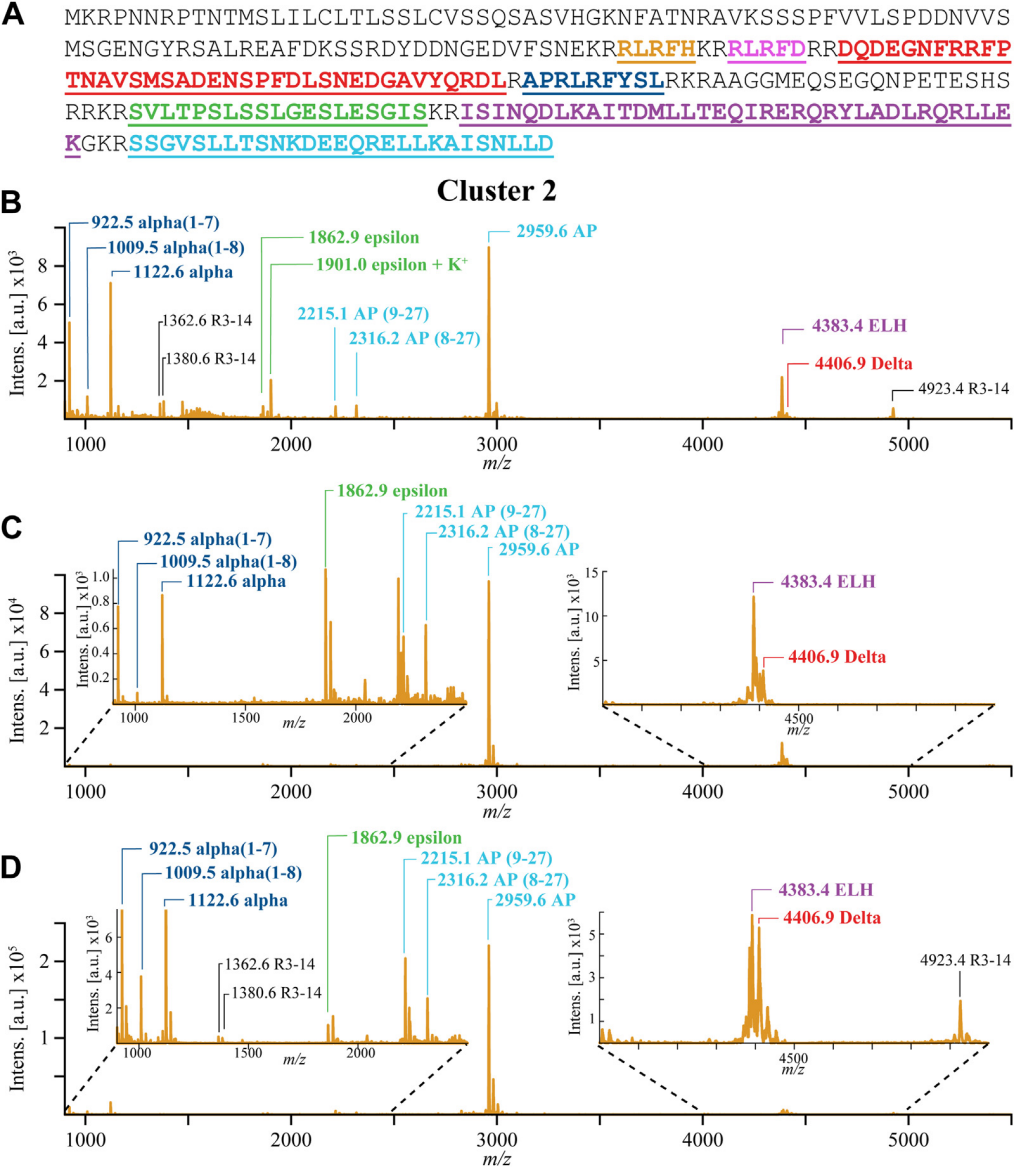
**High-throughput MALDI mass spectrometry analysis of BCNs.** Mass spectra peak annotations include peptide monoisotopic *m/z* and the peptide name. *A*, the sequence of *Aplysia* ELH (ELH, P01362) preprohormone. Sequences of known peptides formed during its processing are color coded. *Orange*, beta-BCP; *pink*, gamma-BCP; *red*, delta-BCP; *dark blue*, alpha-BCP; *green*, epsilon-BCP; *purple*, ELH; *light blue*, acidic peptide. *B*, the annotated average mass spectrum of cells from LJ cluster 2. Cells from this cluster have the greatest ELH prohormone peptide coverage of any LJ cluster found in this study. *C* and *D*, examples of mass spectra acquired from manually isolated single BCNs. The same ELH peptide signals are present in both the average mass spectrum and the mass spectra obtained from manually isolated and individually sampled single BCNs. BCN, bag cell neurons; BCPs, bag cell peptide; ELH, egg laying hormone; LJ, Louvain–Jaccard.

A well-studied example of a single prohormone’s multiple peptides being detected in the same neuron is that of egg laying hormone (ELH, P01362). The ELH prohormone is found in BCNs located in two cellular clusters adjacent to the pleuroabdominal connectives in the vicinity of the abdominal ganglion. ELH prohormone processing (63) produces numerous peptides, including ELH, acidic peptide, and alpha-, beta-, delta-, gamma-, and epsilon-bag cell peptides (BCPs) (Fig. 4*A*), some of which are involved in egg laying behavior in *Aplysia*. (56, 73, 77, 78) In previous studies, single BCNs have been shown via MS to contain and release multiple ELH prohormone peptides (63, 79, 80, 81, 82). Many of these well-characterized ELH peptides have been detected in this study.

LJ cluster 2 (Fig. 3) contains more BCNs than any other LJ cluster (Figs. S2 and S4). The average mass spectrum from these cells was compared with mass spectra from manually isolated and individually sampled BCNs to determine if the high-throughput single cell MS method used here could produce mass spectra of comparable chemical information. Signals for all mature ELH peptides, including several truncated forms, are present in the average mass spectrum of LJ cluster 2 (Fig. 4*B*). Two ELH peptides, beta-BCP and gamma-BCP, have molecular weights below 900 Da and so are outside the mass range of this study. LJ cluster 2’s coverage of the ELH prohormone compares well with manually isolated single neuron mass spectra (Fig. 4, *C* and *D*) and reports from previous studies (63, 79). The approach used here offers high-quality neuropeptide detection in single cells but at a much higher throughput than manual single cell MALDI MS measurements (51, 52, 83, 84, 85).

In addition to chemical fidelity, this high-throughput profiling method demonstrated remarkable sensitivity, detecting peptides potentially originating from cellular terminals. While peptides encoded by the R3-14 prohormone (R01364) were observed in spectra from the BCNs (Fig. 4*B*), the prohormone is known to be expressed in large, identified neurons of the neighboring abdominal ganglion that project into BCN clusters (49, 57). Therefore, R3-14 peptide signals seen in the average mass spectrum of LJ cluster 2 are likely due to the localization of fine terminals of R3-14 neurons innervating or passing through the BCN clusters (72). Such terminals may remain attached to neurons even after the isolation of individual cell bodies. The same R3-14 peptide signals can be seen in the mass spectra of manually isolated BCNs (Fig. 4*D*) and have been found in releasates collected near the bag cell clusters (75).

BCNs are located upon the pleuroabdominal connective and adjacent to the peptidergic R3-R13 neurons and so this could be considered a “worst case scenario” for the colocalized detection of peptides not produced by the isolated cell being detected. The isolation of neighboring cells’ processes along with a sampled cell may be a stochastic event, but it also depends on the levels of peptide present in the process (as well as the detectability of their peptides). For example, in the case of the R3-14 peptides, the cells produce extremely high levels of readily detectable peptides. While this does occur in this case, it may occur in other LJ clusters. However, we expect that the LJ clustering should be minimally influenced by peptides that are detected but that originate outside a cell. This can be seen in LJ cluster 2 as R3-14 peptides are only seen in less than a quarter of the LJ cluster 2 cells (Fig. 2) and so are not defining peptides of LJ cluster 2. This is in comparison with multiple ELH peptides that are found in > 95% of LJ cluster 2 cells.

## Discussion

### Mapping prohormone localization by MS

**Figure 5:**
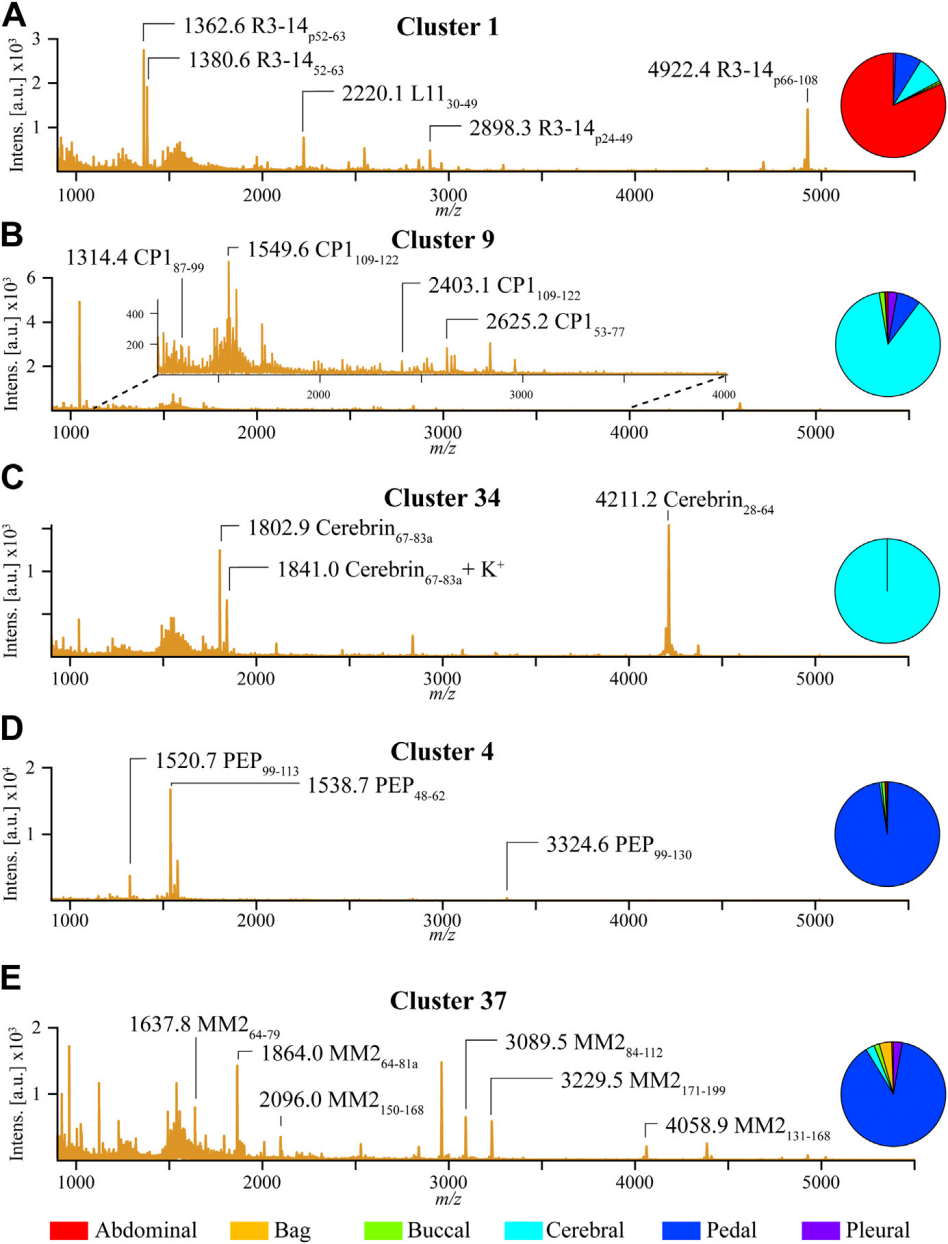
**Average mass spectra and ganglia localization of cells represented by several individual Louvain–Jaccard clusters.** Mass spectra annotations include peptide monoisotopic *m/*z and the peptide’s prohormone name. Insets: pie charts showing what percentage of cells in each cluster come from each ganglion. *A*, LJ cluster 1 average mass spectrum; a primarily abdominal neuron containing LJ cluster defined by L11 and R3-14 peptides. *B*, LJ cluster 9 average mass spectrum; a primarily cerebral neuron containing LJ clusters defined by cerebral peptide 1 (CP1) peptides. *C*, LJ cluster 34 average mass spectrum; an exclusively cerebral neuron containing LJ cluster defined by cerebrin peptides. *D*, LJ cluster 4 average mass spectrum; a primarily pedal neuron containing LJ cluster defined by pedal peptide (PEP) peptides. *E*, LJ cluster 37 average mass spectrum; a primarily pedal neuron containing LJ cluster defined by myomodulin 2 (MM2) peptides.

An advantage of the *Aplysia* neurobiological model is the vast existing knowledge on the localization of many prohormones or their mRNA to specific ganglia within the CNS that has been obtained by immunohistochemistry (IHC), immunocytochemistry (ICC), *in situ* hybridization (ISH), Northern blot, and mass spectrometric approaches. Overall, the distribution of assigned prohormones and related peptides found in this study (Fig. S5) agree with the results of previous works, as seen in several examples shown in Figure 5.

As mentioned previously, the R3-14 prohormone is primarily expressed in abdominal ganglion neurons (49, 57). LJ clusters 1 and 30 (Figs. 3 and 5*A*) all are defined by the presence of R3-14 related peptides and are comprise 82% and 76% abdominal ganglion cells, respectively. LJ cluster 1 is also the only LJ cluster comprising cells containing peptides from the prohormone L11 (P06518). As with R3-14, L11 has been localized to the abdominal ganglion using Northern blot and IHC (86). Consistent with these data, the colocalized detection of peptides from these two prohormones is found in LJ cluster 1, which has the greatest percentage of and contains more abdominal ganglion cells than any other LJ cluster (Fig. S4).

Single ganglion localization is seen with other ganglia and LJ clusters as well. Neuropeptide profiles unique to the cerebral ganglion define LJ clusters 9 (Fig. 5*B*) and 34 (Fig. 5*C*). Both LJ clusters are composed primarily of cerebral ganglion cells (87% and 100%, respectively). LJ cluster 9 comprises cells containing cerebral peptide 1 peptides, and the cerebral peptide 1 prohormone (Q10998) is known to be expressed in the cerebral ganglion (64). Likewise, cerebrin mRNA has been found most abundantly in the cerebral ganglion (51), and the cerebrin prohormone (Q8T112)-related peptides define LJ cluster 34. Pedal peptide (Q5PSJ2) prohormone-related peptides and myomodulin 2 mRNA have been found overwhelmingly in the pedal ganglion (62, 87). LJ clusters 4 (Fig. 5*D*), 27, and 31 are defined by the presence of pedal peptides and contain 97%, 97%, and 99% pedal ganglion cells, respectively. LJ cluster 37 (Fig. 5*E*) is the only LJ cluster whose peptide profile contains myomodulin 2 (Q2VF17) peptides and, not surprisingly, is made up of 89% pedal ganglion cells.

Although the preceding examples are of prohormones and their related peptides being found exclusively in a specific ganglion, some *Aplysia* prohormones are widely distributed throughout the CNS. HPLC and ICC revealed that small cardiac peptides (SCPs) (P09892) are most abundant in the buccal ganglion, with weaker immunostaining observed in disparate neurons in the cerebral, pedal, and pleural ganglia and no staining found in the abdominal ganglion in adult animals (47). In this work, SCPs are most predominant in the peptide profiles of LJ clusters 8, 18, and 23 (Fig. 3). These LJ clusters follow the same trend as described in previous work (47), containing greater than 50% buccal cells; 5% to 30% cerebral, pedal, and pleural cells; and <1%, 8%, or 5% abdominal cells (Fig. S4).

Sensorin A is another prohormone found throughout the *Aplysia* CNS, and whole-mount ISH experiments found sensorin A mRNA in greater than 200 cells in each of the buccal, cerebral, and pleural ganglia; approximately 100 abdominal cells; and only 10 pedal neurons (88). Similar results are seen in this work. The highest sensorin A–containing LJ clusters are 3, 7, 18, 25, and 33. The three largest of these LJ clusters (3, 7, 18) are primarily composed of buccal and pleural neurons, a moderate percentage of cerebral and abdominal neurons, and virtually no pedal neurons (Fig. S4). Interestingly, LJ cluster 33 contains a large proportion of abdominal neurons (46% abdominal versus 26% buccal and 11% pleural). This is perhaps due to the colocalization of sensorin A peptides with L5-67 (P07712) peptides. The L5-67 prohormone has been reported as being localized to the abdominal ganglion (89, 90) and so its colocalization with sensorin A peptides would likely occur in abdominal ganglion cells. LJ cluster 25 comprises a majority (54%) of pedal neurons, an interesting result given that these cells colocalize sensorin A and apSPTR-GF-DP (Q5PSJ3), which IHC analysis found mostly in cerebral cell soma, but did find extensive staining in pedal ganglion fibers and neuropile (53).

### Single Neuron colocalization of prohormones

**Figure 6:**
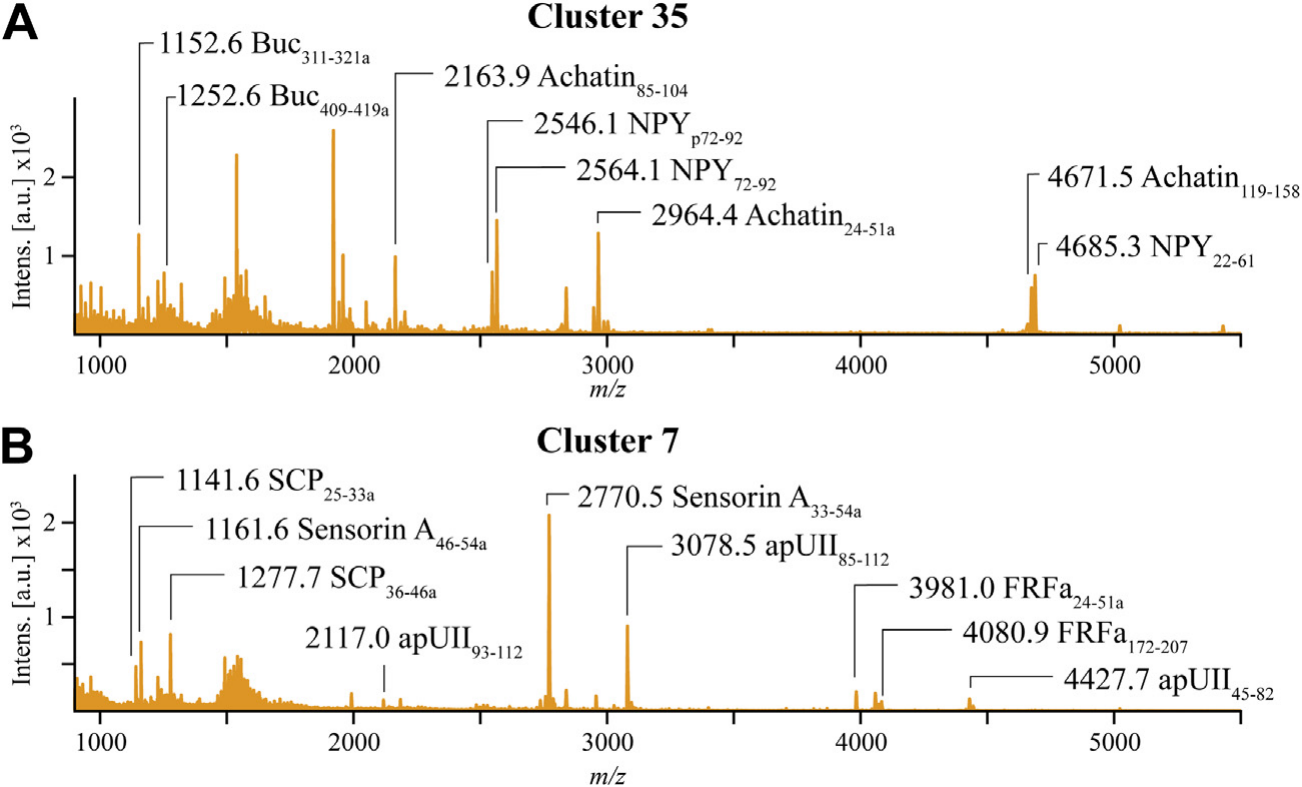
**Coexpression of multiple prohormone-related peptides in the average mass spectra of all cells within their respective Louvain–Jaccard clusters.** The different prohormones seen in each mass spectrum have been previously shown to colocalize in individual neurons (59, 61). The peptides colocalize in neurons that form distinct Louvain–Jaccard clusters out of all of the 26,797 *Aplysia* cells sampled, shown here for (*A*) Cluster 35 and (*B*) Cluster 7. Mass spectrum annotations include peptide monoisotopic *m/*z and the peptide prohormone names.

Cotransmission increases a neural circuit’s plasticity by the simultaneous or differential release of signaling molecules that can act on different targets by activating different receptors (7, 91). Some of the most diverse LJ clusters in this work are defined by cellular colocalization of peptides from behaviorally related prohormones. LJ cluster 35 (Figs. 3 and 6*A*) contains cells expressing peptides from achatin (Q5MAR6), neuropeptide Y (NPY) (Q27441), and buccalin (P20481). This cluster exhibits the same combination of achatin and NPY peptides as previously shown by MS analysis of manually isolated single neurons: achatin peptides (monoisotopic *m/z*: 2163.9, 2964.4, 4671.5) and NPY peptides (monoisotopic *m/*z: 2546.1, 2564.1, 4685.3) (59). That same work also showed the achatin prohormone-derived peptide GdFFD as an extrinsic modulator of the feeding network. NPY has also been shown to be involved in feeding. The injection of the peptide Aplysia neuropeptide Y (apNPY) (monoisotopic *m/z* 4685.3, NPY_22-61_) reduces food intake and slows the rate of ingestion by *Aplysia*. NPY release was also measured from stimulated feeding network neurons (92). While not previously reported in single cell mass spectra with achatin and NPY, buccalin peptides are known to influence similar behaviors, including activity in the accessory radula closer (ARC) muscle that is responsible for biting (58, 93). The feeding neuronal network mediates complex and diverse feeding behavior with sensory, decision-making, and locomotor components. Key neurons within this circuit are responsible for specific and well-defined tasks and may have different neurochemical toolkits serving different feeding behavior components (94).

In addition to the behavioral relationships, the results from this work are also consistent with ganglion localization studies of buccalin, NPY, and achatin. ISH staining found buccalin mRNA primarily in the cerebral and pedal ganglia and abdominal nerves (58). ICC and ISH analyses have shown that NPY appears to be distributed across the CNS, with the most prominent mRNA expression in the pedal ganglion (63, 92, 95), and ISH showed strong achatin mRNA expression in the pedal ganglion with little or no staining in other ganglia (59). The cellular population of LJ cluster 35 is >90% pedal cells (Fig. S4) and consistent with these ganglia localizations.

LJ cluster 7 represents a neuronal population that contains SCP, sensorin A, urotensin II (apUII, XP_012945633.1), and FRFa (S2XV90) peptides (Fig. 6*B*). Peptides from apUII have shown activity in the *Aplysia* feeding network (52). That study also used MS to show single cells colocalizing apUII peptides (monoisotopic *m/z*: 2117.0, 3078.5, 4427.2) with multiple FRFa peptides, which was also observed here (Fig. 6*B*). FRFa peptides are also active in the feeding network as well as being found in the ARC muscle (61).

In addition to apUII and FRFa, LJ cluster 7 cells also contained SCP and sensorin A peptides. SCPs have been shown to modulate the ARC muscle during feeding (96). Sensorin A is found in buccal mechanosensory neurons whose receptive fields include the perioral lip region (88). MS measurements of individual mechanosensory buccal S neurons have demonstrated the colocalization of the following peptides: sensorin A and B (monoisotopic *m/*z 1161.6 and 2770.5), SCPa and SCPb (monoisotopic *m/*z 1141.6 and 1277.7), and numerous FRFa peptides (including monoisotopic *m/z* 3981.0 and 4080.9) (61). Similar peptide colocalization was observed in this study (Fig. 6*B*). The colocalized detection of sensorin A, SCP, and FRFa in buccal S neurons is consistent with the results in this work where 90% of LJ cluster 7 cells are from the buccal ganglion (Fig. S4).

Characterization of the neuropeptide content of the CNS is one key component in understanding a neuron’s function, both individually and within larger neural circuits. This work describes the single cell peptide heterogeneity of neuronal populations from the major CNS ganglia of the neurobiological model organism *A*. *californica*. Methodological advances allowed for the high-throughput MS sampling and subsequent LJ clustering of over 26,000 single neurons ranging in size from 10 to 300 μm. The global and high-throughput approach produced mass spectral peptide profiles similar to those reports discussed above of the MS characterization of manually isolated single neurons. Importantly, the results described here are in good agreement with and expand upon previously published data on known prohormone processing, ganglia localization, and single cell peptide colocalization in the *Aplysia* CNS and individual neurons.

In terms of potential limitations of the approach, two are worth mentioning. First, we used the most common MALDI mass analyzer, the TOF, as it demonstrates that the approach can be used with equipment found in facilities at most universities. However, this limits the mass resolution and MS/MS abilities (for high-confidence peptide assignments); if higher-quality spectra are required or peptide sequencing is needed, Orbitrap (10, 12), ion cyclotron resonance (40, 41, 42), or timsTOF (97, 98) instruments are also amenable to the sampling and informatics workflow. Second, while this protocol is effective at detecting the peptides within cells, a mass spectrometer will measure whatever is at the specific location on the slide. The fidelity of the single cell data depends on the ability to dissociate the tissue into cells. For example, if the cells have terminals from other cells attached, or other material from distinct cellular origins, the spectra will have features in it from more than one cell. One expects that, for certain cell types or at some locations, the presence of vasculature, nerves, or other features may impact the quality of cell isolations and so sampling bias may be introduced. Thus, it is important to carefully image and examine the cell preparations to ensure the cells are distinct and well separated.

Overall, these results complement information obtained by single cell transcriptomics, manual single cell isolation MS analysis, and tissue-level peptidomics. Although we demonstrate our single cell protocols using *Aplysia*, this workflow is well suited to studies using animals that may not have the same level of biochemical and functional information available. The projected output will guide new functional and behavioral studies of individual neuropeptides, aid the understanding of the functional relationship between neuropeptides and neuronal networks, and provide an overview of neuropeptide and hormones with other animal models.

## Experimental procedures

### Animals, single cell isolation, and sample preparation

Late juvenile and adult *Aplysia*, 120 to 200 g body weight, were purchased from the National Resource for *Aplysia*. Animals were anesthetized using a 50% w/v injection of 14 °C isotonic (390 mM) MgCl_2_ solution into the body cavity. The CNS was surgically isolated and stored in artificial sea water (ASW) containing in mM: 460 NaCl, 10 KCl, 10 CaCl_2_, 22 MgCl_2_, 6 MgSO_4_, and 10 Hepes at pH 7.8. The CNS was enzymatically treated for 90 min at 34 °C in ASW solution containing 10 mg/ml protease IX, 100 units/ml penicillin G, 100 μg/ml streptomycin, and 100 μg/ml gentamicin. The enzymatic treatment reduced the mechanical integrity of the ganglionic sheath and the connections between individual cells, which assisted single cell isolation. After enzymatic treatment, CNS ganglia were rinsed in fresh ASW, surgically separated, and desheathed and nerves and connective tissues were removed. Isolated, whole ganglia were stained using 10 μg/ml Hoechst 33342 in ASW for 30 min and then stabilized in 33/67 glycerol/ASW solution for 5 min.

The glycerol/ASW stabilized bag cell clusters and abdominal, buccal, cerebral, pedal, and pleural ganglia were transferred to conductive indium tin oxide–coated glass slides (Delta Technologies) with etched fiducial markers. Ganglia were mechanically manipulated, causing single cells to be distributed across the slide at low density. To reduce batch effects, each slide contained six regions of interest (ROIs) and each ROI contained cells from a randomly selected ganglia and animal. After cell dispersal, slides were stored overnight to allow the cells to adhere and then quickly rinsed with 1 ml of 150 mM ammonium acetate before analysis. In total, 18 animals were sacrificed across 6 days and cells were spread across 54 slides.

Each slide was optically imaged in brightfield and fluorescent channels using a Zeiss Axio Imager M2 microscope (Zeiss) equipped with an AxioCam ICc5 camera, X-cite Series 120 Q mercury lamp (Lumen Dynamics), and a HAL 100 halogen illuminator (Zeiss). The DAPI filter set (excite: 335–383 nm, emit: 420–470 nm) was used for the fluorescent imaging. Imaging with a 10 × objective resulted in 0.55-μm/pixel spatial resolution. Individual images were acquired and stitched together with a 12% overlap. Stitched images were exported in BigTIFF format using Zen software version 2 blue edition (Zeiss). Using microMS (36), single cells in each ROI were identified and their slide coordinates recorded. Objects identified by microMS were filtered based on shape and size to remove nonneuronal material such as sheath, glia, lysed cells, and other debris. To ensure only single cells were sampled, cells were distance filtered so that no two cells within 200 μm were sampled. The remaining predominately cellular structures in each ROI had their coordinates converted to stage motor positions via microMS, and these positions were exported as xeo geometry files for automated instrument acquisition.

Once optically imaged, slides had a 50 mg/ml 2,5-dihydroxybenzoic acid solution in 1:1 water:ethanol and 0.1% TFA applied using an artist’s airbrush. Slides were weighed before MALDI matrix application and after matrix drying. A 1.20 ± 0.25 mg/cm^2^ (mean ± standard deviation) dihydroxybenzoic acid coating was applied to each slide.

### Single cell mass spectrometry

Single cell mass spectra were acquired using an ultrafleXtreme II MALDI-TOF/TOF mass spectrometer (Bruker Corp) using positive reflectron mode and an *m/z* window of 480 to 5500, externally calibrated using Bruker Pep Mix II and bovine insulin. Each mass spectrum was acquired using 1000 laser shots at 1000 Hz and a 100-μm-diameter footprint using the “ultra” laser setting. microMS-generated xeo geometry files were used to automate data acquisition in each ROI. Since many of the targeted *Aplysia* neurons were larger than the 100-μm laser footprint, and MALDI matrix crystallization did not always occur atop these larger cells, four mass spectra were acquired at different points centered on the edge of each cell. To reduce experimental errors, the slides, ROIs, and cells within each ROI were sampled in a random order.

### *In silico* peptide library compilation

Known *Aplysia* prohormones were retrieved from UniProt database by searching with the keywords “Aplysia peptide” and “Aplysia prohormone.” Hits from both searches were downloaded, combined, and manually curated for duplicates; proteins with the word “receptor” or “enzyme” in their names and proteins >1000 amino acids long were excluded. The search included both “reviewed” and “unreviewed” entries. A total of 72 unique proteins were batch processed for the presence of a signal peptide using the SignalP 5.0 tool (99). The *in silico* neuropeptide library was computed via the NeuroPred (100, 101) prediction tool, with the “Mollusc” model; PTM settings included amidation and pyroglutamylation, and the possibility of a disulfide bond was manually inspected in peptides with two cysteine residues. Peptides predicted in this way were compiled to form a putative peptide library.

### Mass spectrometric data processing

Raw mass spectra were exported as text files from flexAnalysis (Bruker Corp) and imported into MATLAB 2017a (The Mathworks Inc). The four replicate mass spectra from each cell were summed into a single representative mass spectrum. Each mass spectrum’s baseline was calculated using the MATLAB msbackadj function. The noise at each *m/z* was calculated as the standard deviation of signals below the 99th percentile within a 200-*m/z* window around each *m/z*. Mass spectral peaks were found using the mspeaks function and filtered to only include monoisotopic masses. Peaks were only kept if they had an S/N greater than five.

To correct for instrumental drift and possible losses in slide planarity, the single cell peak lists were aligned and then recalibrated as follows. Each peak list was aligned to the *m/z* value of one of several known, abundant peptide signals detected in the cell’s respective ganglion. If no peak was present in the single cell peak list with a mass error less than 200 ppm, the peak list was aligned using a lipid *m/*z. If no lipid *m/z* was found in the peak list with a mass error of less than 200 ppm, the peak list was removed from further analysis. The list of known, abundant peptide and lipid *m/z* values used to align mass spectra from each ganglion are available in the supporting information (Table S2).

The aligned single cell peak lists in each ROI were combined into a single data set. Each ROI was independently recalibrated using a quadratic fit to known, abundant peptide signals in the ROI’s respective ganglion. Only peaks with a mass error of less than 75 ppm were used in the recalibration process. The information on peptide calibrants for each ganglion can be found in the supporting information (Table S3). The signals in each single cell peak list were then assigned to peptides based on matching, with a mass error of less than 60 ppm, to an *in silico* library of 1333 *Aplysia* peptides generated as described above. Assigned peptides from the single cell mass spectra were examined for a peptide mass fingerprint of prohormones; if only one peptide from a given prohormone was assigned to a signal in a single cell spectrum, that peptide was removed from the assigned peptide list. If a prohormone in the library produced fewer than four peptides, singular peptide assignment from that prohormone was allowed. The single cell assigned peptide lists then had any assigned isobaric peptides removed from the lists. The single cell assigned peptide lists were then checked a second time and any singularly assigned peptides that may have resulted due to isobar removal were also removed from the lists.

### LJ cell clustering

After single cell profiles were established, LJ clustering (102) was used to categorize cells based on their respective peptide profiles. LJ clustering has previously been shown to robustly categorize single cell transcriptomic (103) and single cell mass spectrometric data (38). In this analysis, the matrix of each cell’s assigned peptide log_2_ (S/N) (866 peptides by 26,797 cells) was used to construct a KNN graph. For the data collected, the appropriate number of KNNs is not known. Therefore, the appropriate KNN was determined by examining the bootstrapping results. First, LJ clustering was done using all cells 40 times, with KNN 5, 10, 15…200 using the cosine distance metric and peptide Jaccard index for edge weight. Next, 1000 bootstrap experiments were done, with each using a random 75% of all cells to construct a KNN graph with a randomly chosen KNN between 5 and 200. Noise was introduced to the bootstrapped KNN graph by deleting 5% of the edges and adding 5% spurious edges of a random weight between random cell pairs. LJ clustering was then performed on each of the 1000 graphs.

Two measures, stability and purity (103), were used to compare the 40 all-cell LJ clustering outcomes with the 1000 bootstrapped outcomes. Stability describes how well cells from a given cluster *k* in the all-cell outcomes group together in the bootstrapped outcomes. Stability can range from 0 (cluster *k* cells are not grouped together in the bootstrapped outcome) to 1 (cluster *k* cells are present in a single cluster in the bootstrapped outcome). While a high stability value indicates that cluster *k* cells group together after bootstrapping, it does not mean that they group exclusively by themselves. Purity is used to describe the exclusivity, or the extent to which cluster *k* cells group with only cluster *k* cells after bootstrapping. Purity also ranges from 0 to 1, where a lower value indicates cluster *k* cells group with cells from other non-*k* clusters in the bootstrapped outcomes.

MALDI mass spectra are known to display shot-to-shot variability in signal intensity. To minimize the effect of these variations on LJ clustering, three steps were taken: 1) the log_2_ of the peptide S/N was used; 2) nearest neighbors were calculated using the cosine distance; and 3) the edges of the KNN graph were defined by the neighboring cells’ peptide Jaccard index. With these considerations it was still possible that some clusters would differ in a given peptide’s S/N and not the frequency of a peptide’s detection in single cells in the LJ clusters. To test this, the cellular frequency of each peptide in each cluster was compared in a pair-wise manner using a chi-square test of independence and Bonferroni corrected for multiple comparisons. Detected peptides were required to be present in at least 20% of the cells in one LJ cluster to avoid incorrectly rejecting the null hypothesis at low expectation values.

## Data Availability

Research data are available by contacting the corresponding author.

## Supporting information

This article contains supporting information.

## Conflict of interest

The authors declare that they have no conflicts of interest with the contents of this article.

## Supporting information

Chan-Andersen_JBC_2022_SI_final revised
