# Supplementary material for: Profiling 26,000 Aplysia californica neurons by single cell mass spectrometry reveals neuronal populations with distinct neuropeptide profiles
Source: J Biol Chem. 2022 Jul 11;298(8):102254. doi: 10.1016/j.jbc.2022.102254 (PMC9396074; doi:10.1016/j.jbc.2022.102254)
Supplement: Chan-Andersen_JBC_2022_SI_final revised [file mmc1.docx]

**Supporting Information**

Profiling 26,000 *Aplysia californica* neurons by single cell mass spectrometry

Peter C. Chan-Andersen, Elena V. Romanova, Stanislav S. Rubakhin, Jonathan V. Sweedler^*^

Department of Chemistry and the Beckman Institute for Advanced Science and Technology, University of Illinois at Urbana-Champaign, Urbana, Illinois 61801

^*^Corresponding author: [jsweedle@illinois.edu](mailto:jsweedle@illinois.edu)

**Table of Contents**

Figure S1: Cell size distribution…………………………………………………………………S-2

Figure S2: Number of cells per cluster………………………………………………………… S-3

Figure S3: LJ clustering…………………………………………………………………………S-4

Figure S4: Ganglia of origin per cluster…………………………………………………………S-6

Figure S5: Prohormone frequency per ganglion………………………………………………...S-7

Table S1: Major *Aplysia* ganglia and their functions………………………………………......S-11

Table S2: Peptides and lipids used for MS alignment…………………………………………S-12

Table S3: Peptides and lipids used for MS recalibration……………………………………….S-14

**
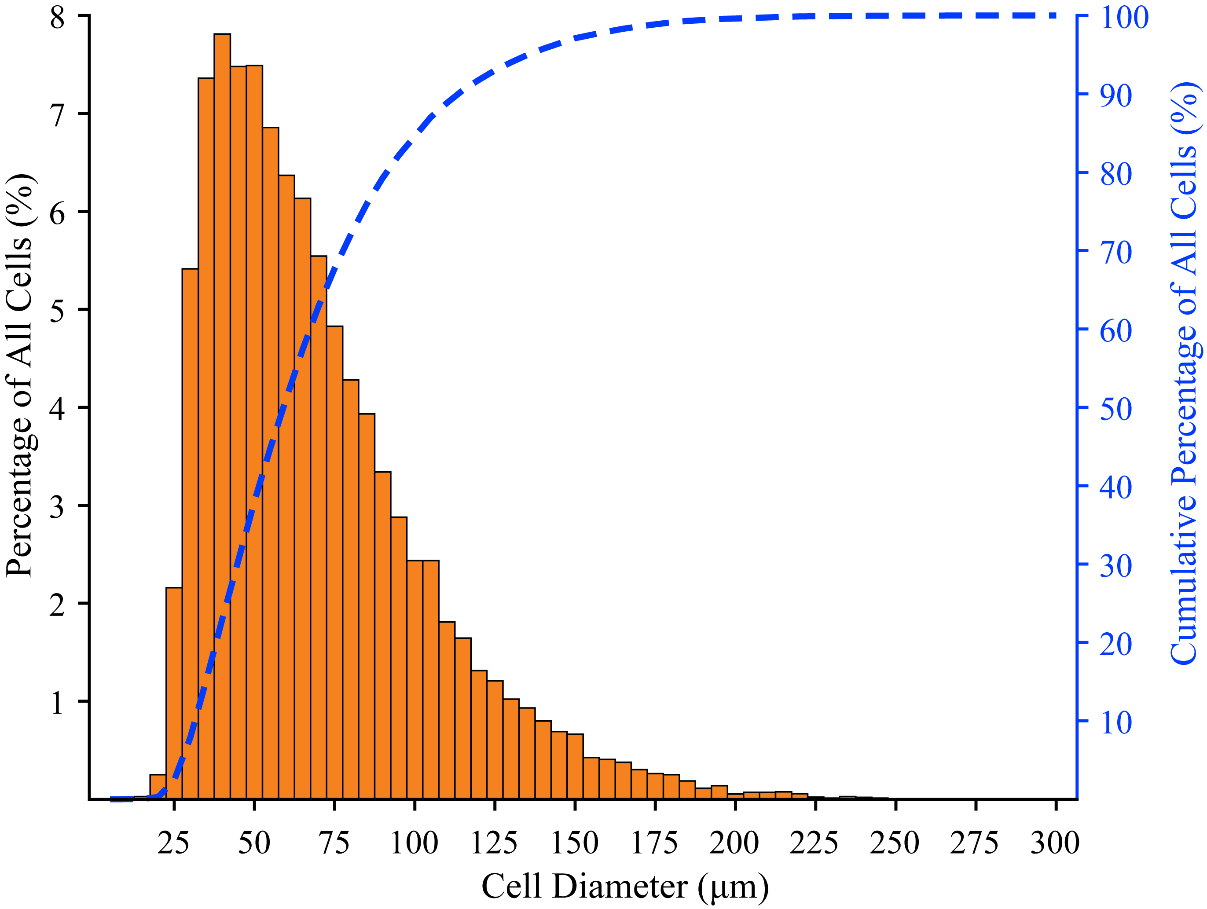
**

**Fig S1.** The distribution of cell diameter in microns of single cells sampled using high throughput MALDI MS. Neurons ranged from 10 to 300 μm and the average size of sampled neurons was 67 ± 34 μm (mean ± standard deviation). The 10^th^, 50^th^, and 90^th^ percentile values are 32, 59, and 113 μm, respectively.


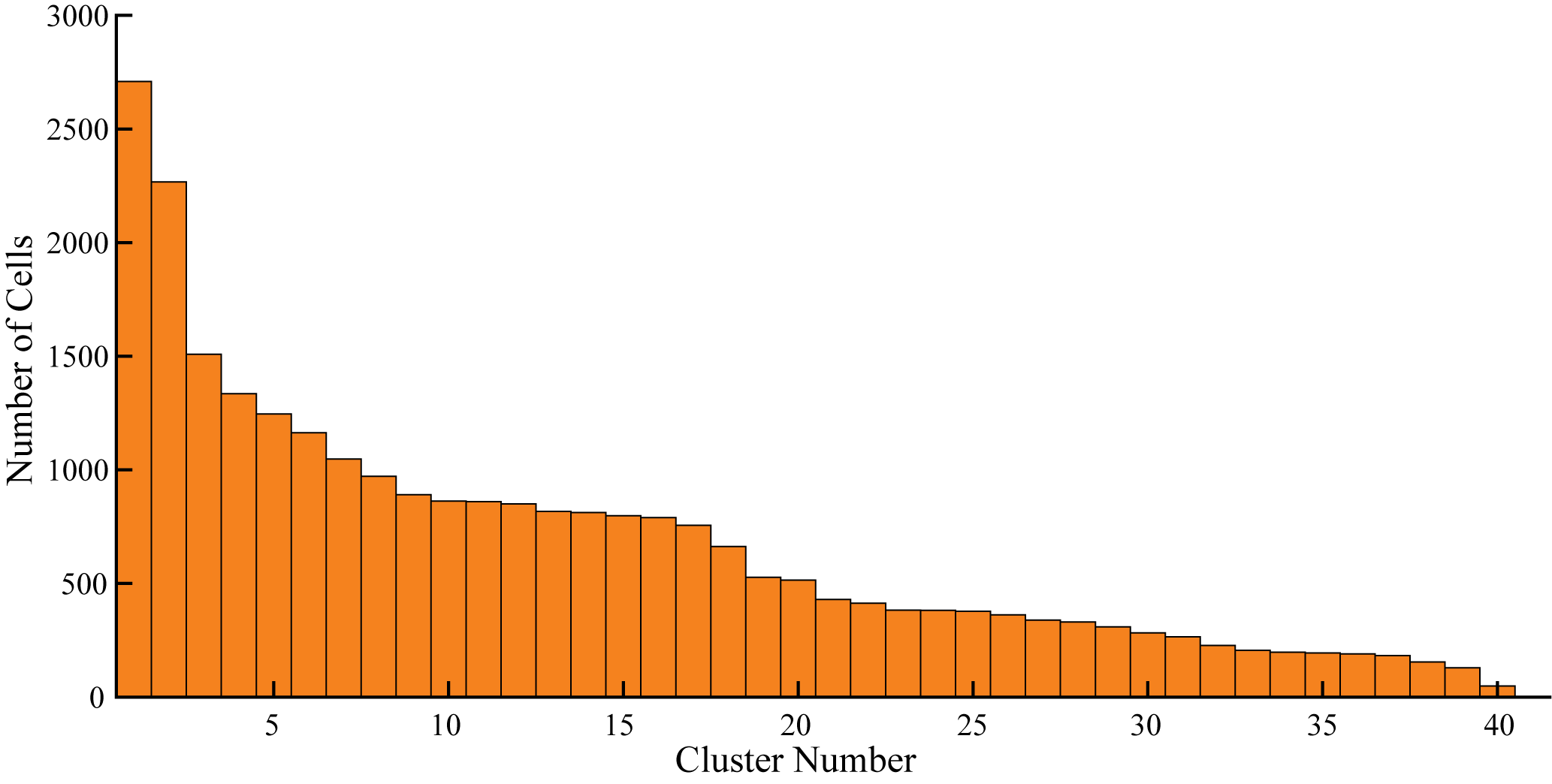


**Fig. S2:** The number of cells in each of the 40 post-merge LJ clusters formed with a KNN of 100. The fewest number of cells in cluster 40 with 49 cells.


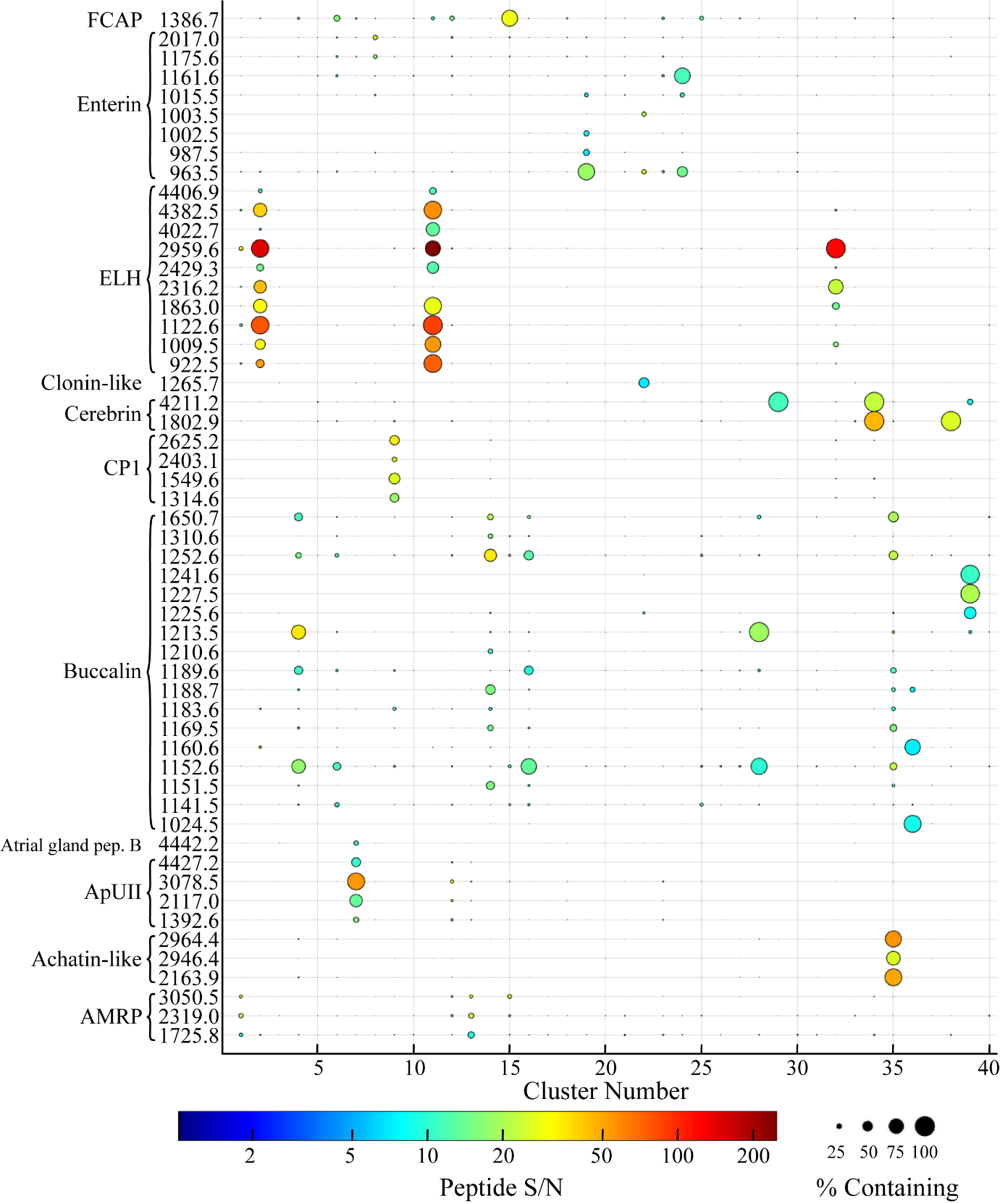


**Fig. S3** continued


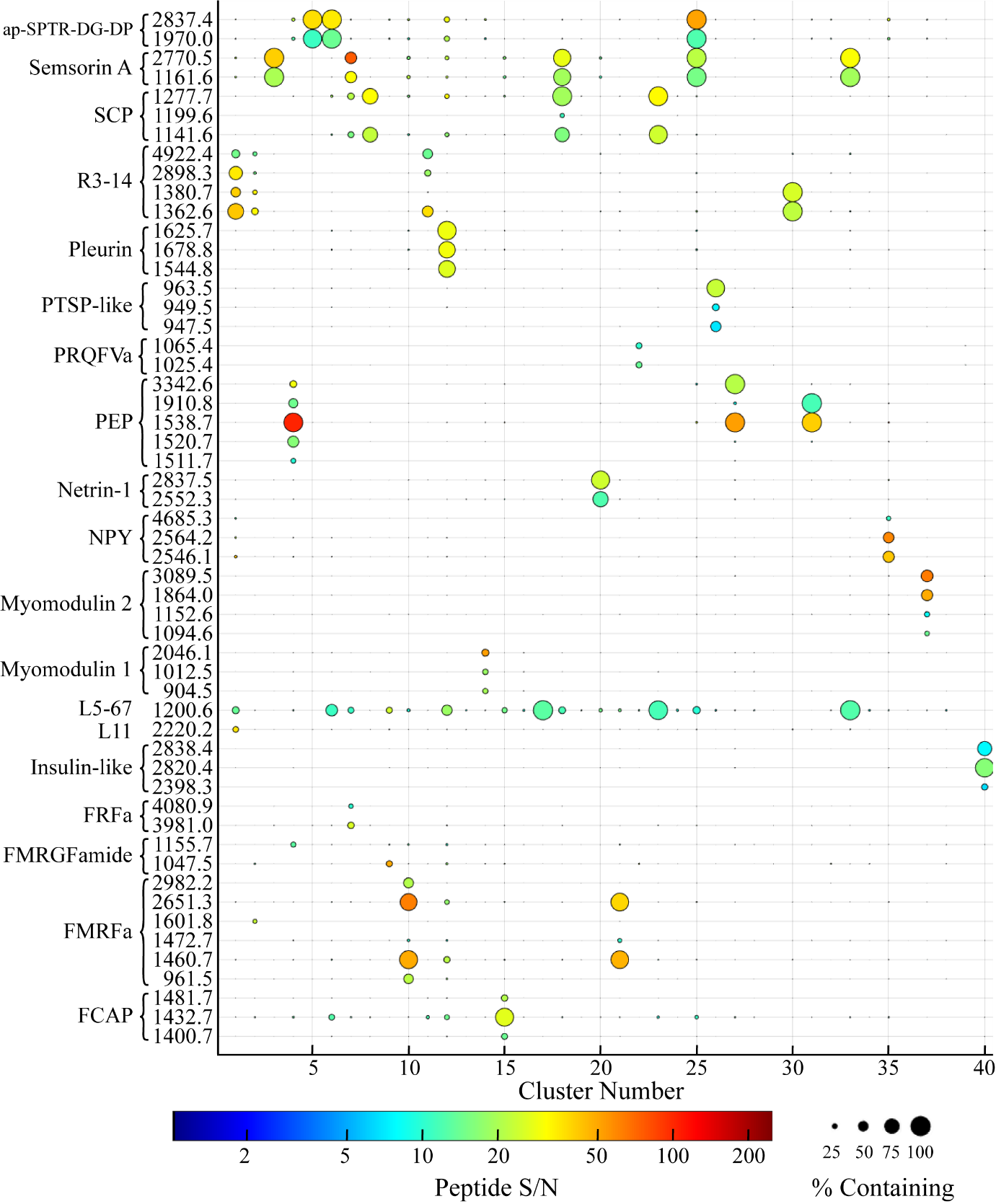


**Fig. S3.** Louvain Jaccard clustering of single *Aplysia californica* neuron mass spectra. LJ clusters were determined using a KNN of 100 and the assigned peptides within each cell. As determined by a chi-square test of independence, a subset of 108 of the 866 assigned *Aplysia* peptides were used in LJ clustering. Each cluster is defined by a unique combination of assigned peptides and/or cellular frequency of their detection. The prohormone name and related peptide monoisotopic *m/z* is given on the ordinate. Circle size corresponds to a peptide’s frequency of detection in each cluster. Color scale depicts the average peptide S/N in a given cluster.


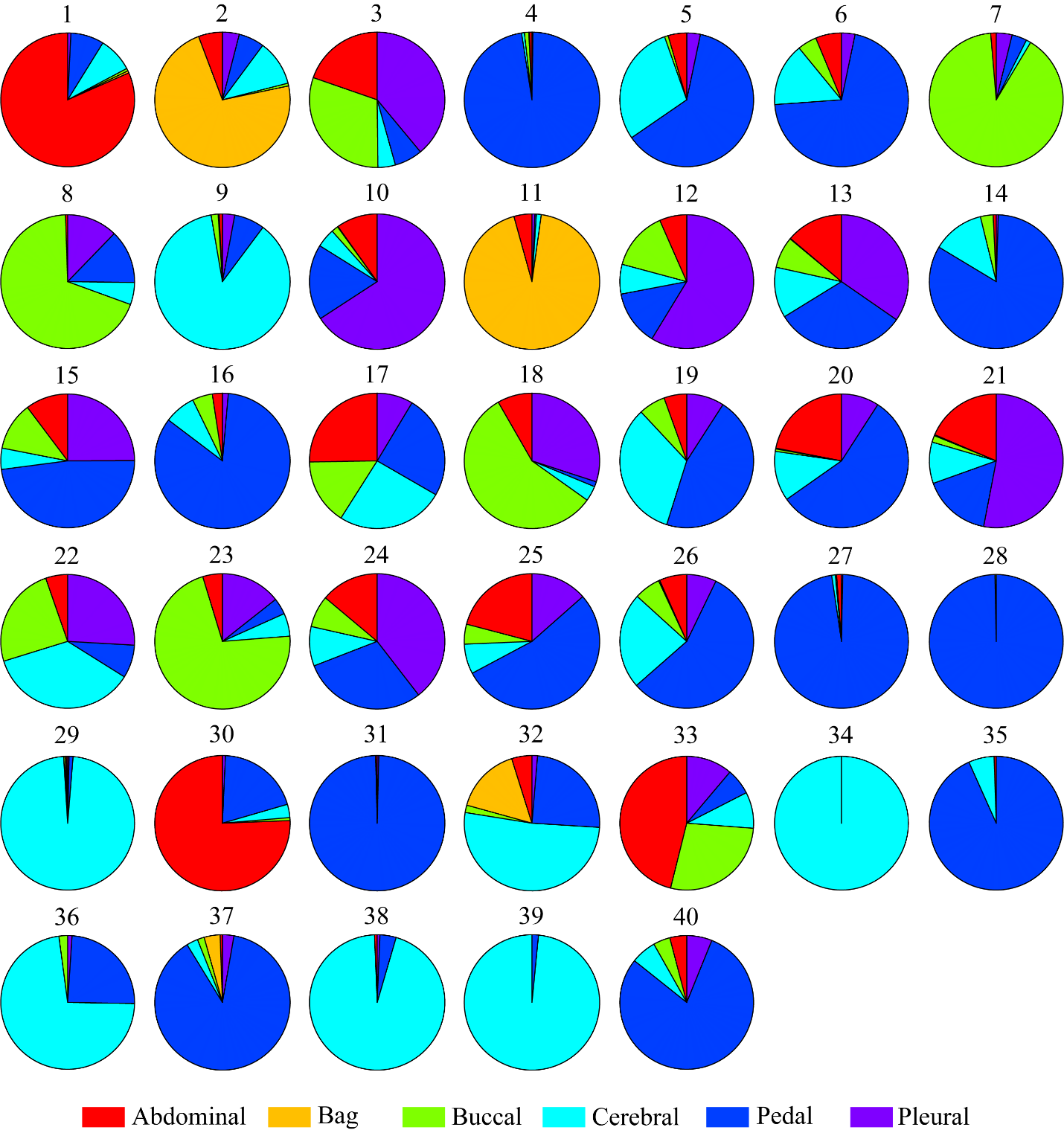


**Fig. S4.** Ganglia of origin for each LJ clusters’ constituent cells. Graphs for all 40 LJ clusters are depicted.


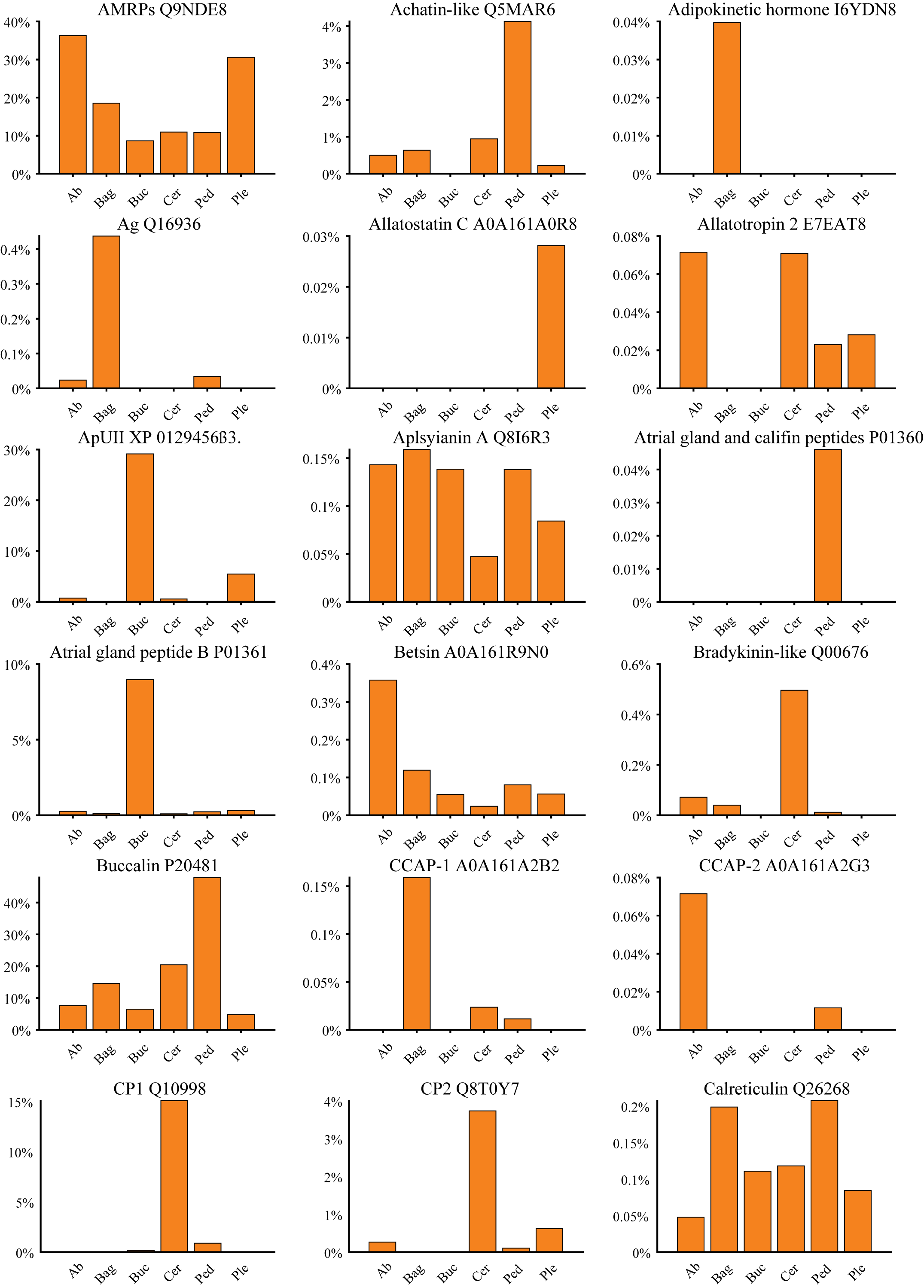


**Fig.S5** continued


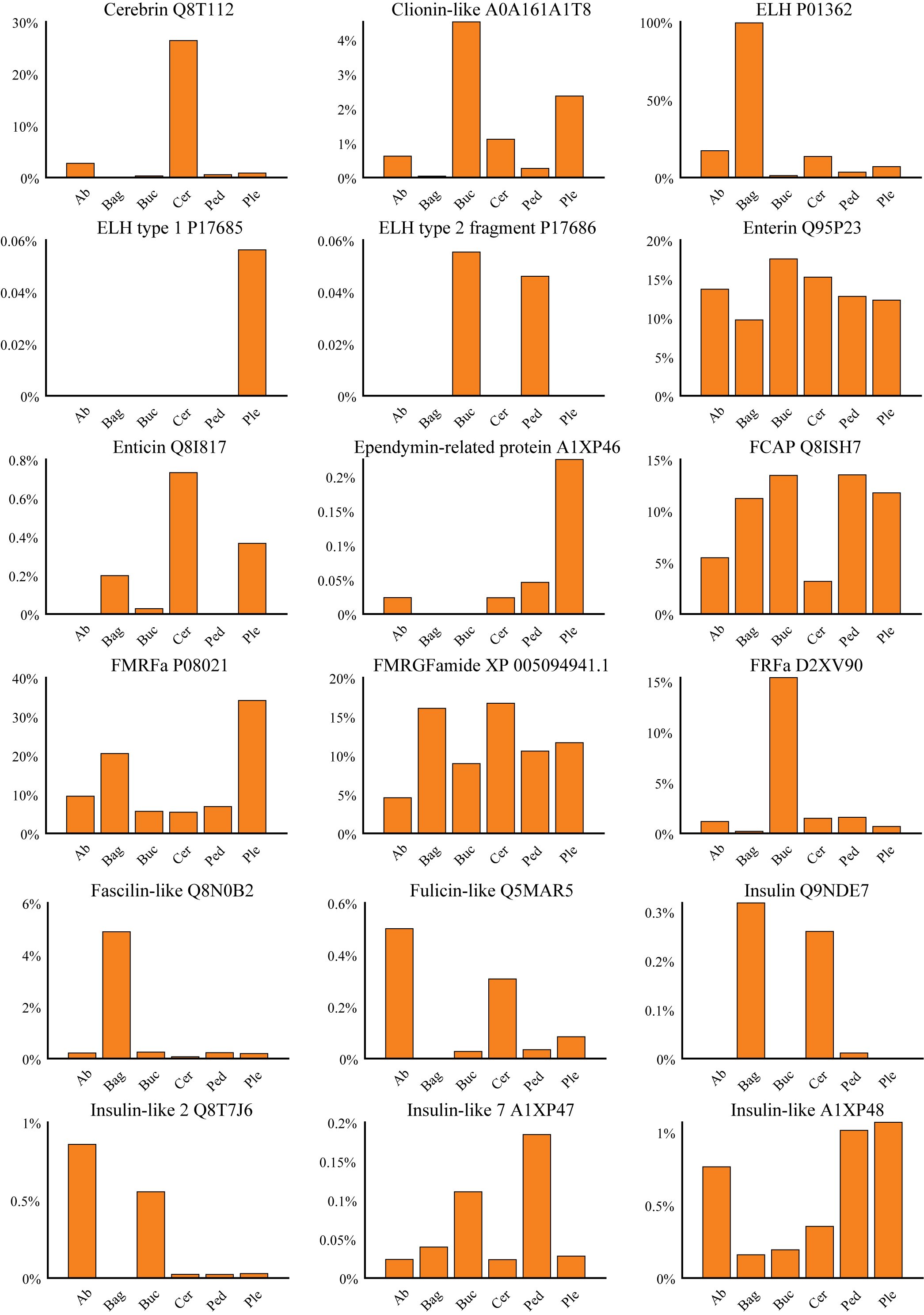


**Fig. S5** continued


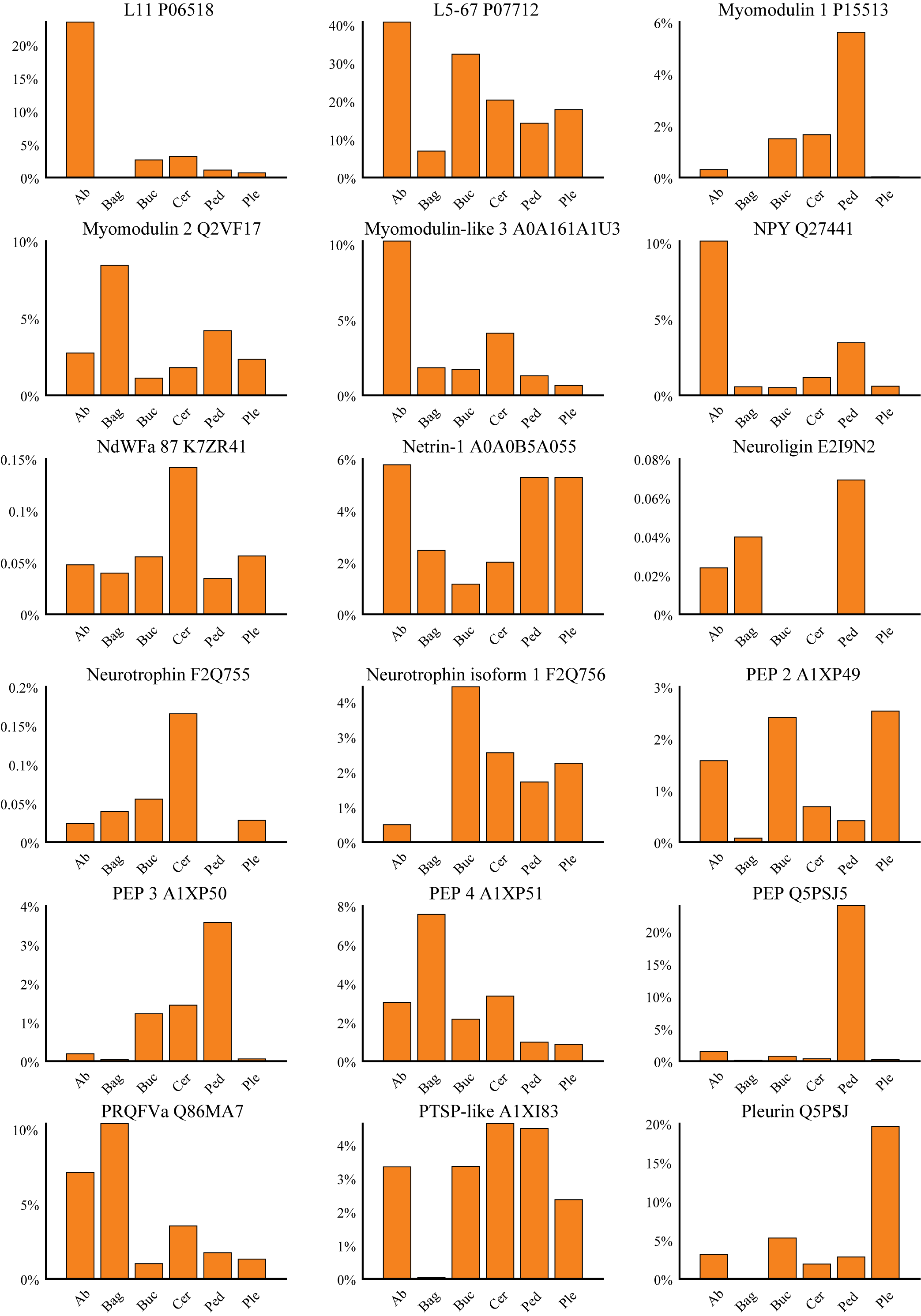


**Fig. S5** continued


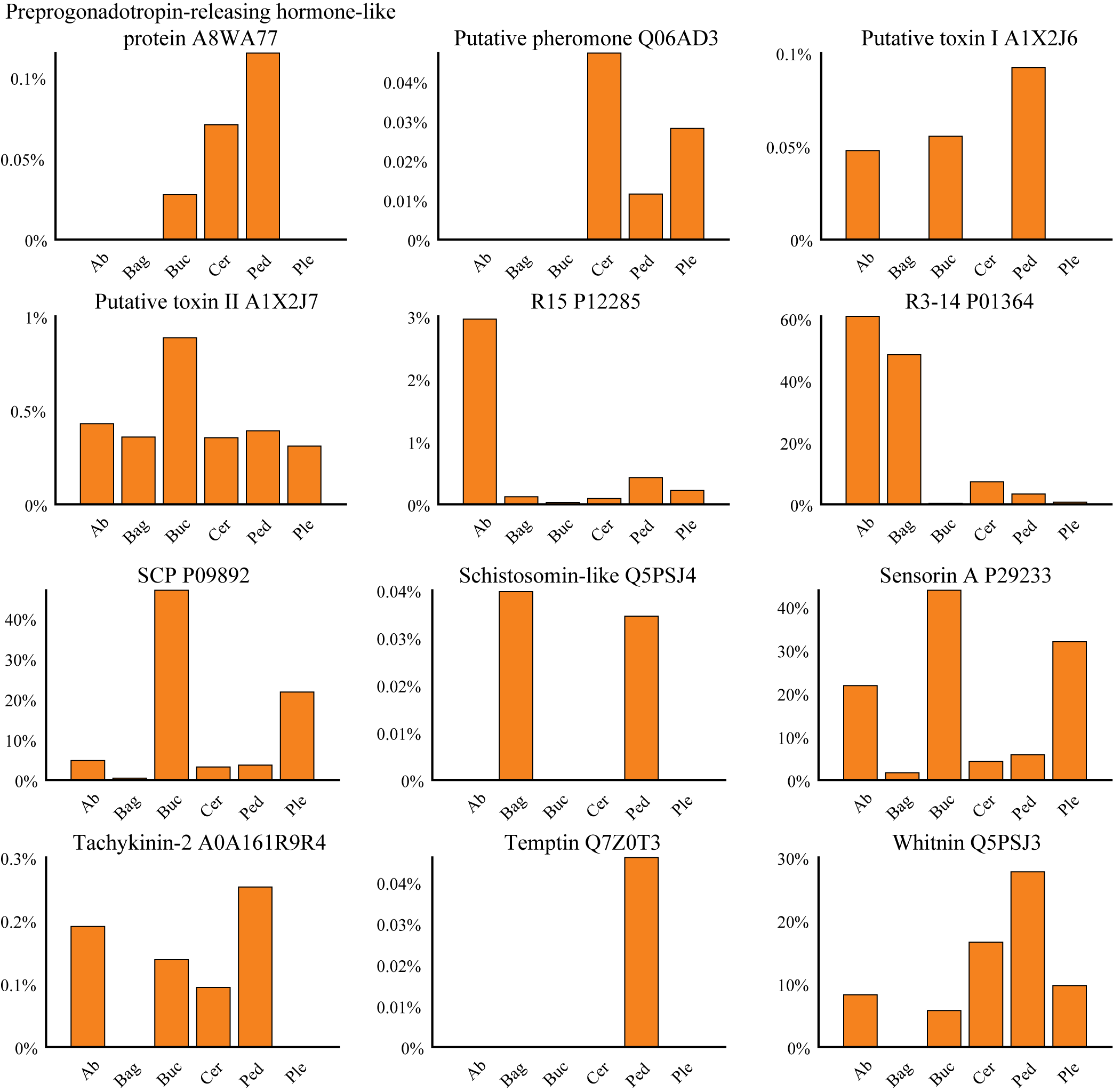


**Fig. S4.** Ganglia-specific abundance of all prohormone-related peptides detected in each ganglion.

**Table S1.** The major ganglia of *Aplysia* and their associated functional and behavioral roles.

| Ganglion | Functions |
| --- | --- |
| Cerebral | Ganglion is primarily involved in sensory functions. Cerebral neurons innervate sensory structures such as the mouth, eyes, and rhinophores. Along with the pedal ganglia, innervates the penis. Also, appears to have some involvement in feeding, appetitive state, locomotion. |
| Buccal | Primary ganglia involved in feeding. Buccal neurons innervate the pharynx, esophagus, crop, gizzard, salivary glands, and muscles of the buccal mass. |
| Pedal | The pedal ganglia are largely involved in locomotion including head movements and swimming. Pedal neurons innervate the foot, head, parapodia, and penis. |
| Pleural | The functions of the pleural ganglia and the innervation of pleural neurons are less well known. |
| Abdominal | The abdominal ganglion largely controls many reproductive, respiratory, circulatory, and excretory functions as well as some defensive movements including siphon and gill withdrawal and ink secretion. The bag cell clusters offer primary control egg-laying and its associated behaviors. |

**Table S2.** Abundant, known peptides and lipids used to align mass spectra from each ganglion. The peptides and lipids are listed in the order in which they were searched for in the spectra. If a spectrum was found to contain a peptide or lipid, it was aligned to that peptide or lipid and the subsequent peptides or lipids were not searched for.

| **Abdominal Ganglion** | | |
| --- | --- | --- |
| Prohormone | Peptide | Monoisotopic *m/z* |
| R3-14 | [p-]EAEEPSAFMTRL | 1362.642 |
| R3-14 | EAEEPSAFMTRL | 1380.652 |
| SCP | ARPGYLAFPRM[Amide] | 1277.701 |
| L11 | RPRIDC(-1.01)TRFVFAPAC(-1.01)RGVSA | 2220.149 |
| apSPTR-DG-DP | MRYMGIC(-1.01)MKKQYNNFIPFPC(-1.01)LRS[Amide] | 2837.373 |
| AMRPs | SSEFPTSNSEQLALDT | 1725.787 |
| L5-67 | APSWRPQGRF[Amide] | 1200.646 |
| NPY | [p-]EFFRTNGERYPEDAAAWTEFQ | 2546.143 |
| R3-14 | [p-]EEVFDDTDVGDELTNALESVLTDFKD | 2898.311 |
| Sensorin A | TRSKNNVPRRFPRARYRVGYMF[Amide] | 2770.514 |
| Sensorin A | ARYRVGYMF[Amide] | 1161.606 |
| NPY | DNSEMLAPPPRPEEFTSAQQLRQYLAALNEYYSIMGRPRF[Amide] | 4685.310 |
| ELH | ISINQDLKAITDMLLTEQIRERQRYLADLRQRLLEK[Amide] | 4382.453 |
| Lipid dimer | (C_42_H_85_NO_7_P)_2_ | 1492.213 |
| Lipid | C_42_H_85_NO_7_P | 746.606 |
| **Bag Cell Clusters** | | |
| Prohormone | Peptide | Monoisotopic *m/z* |
| ELH | SSGVSLLTSNKDEEQRELLKAISNLLD | 2959.564 |
| ELH | SVLTPSLSSLGESLESGIS | 1862.965 |
| ELH | APRLRFYSL | 1122.632 |
| ELH | ISINQDLKAITDMLLTEQIRERQRYLADLRQRLLEK[Amide] | 4382.453 |
| R3-14 | [p-]EAEEPSAFMTRL | 1362.642 |
| R3-14 | EAEEPSAFMTRL | 1380.652 |
| Lipid dimer | (C_42_H_85_NO_7_P)_2_ | 1492.213 |
| Lipid | C_42_H_85_NO_7_P | 746.606 |
| **Buccal Ganglion** | | |
| Prohormone | Peptide | Monoisotopic *m/z* |
| SCP | ARPGYLAFPRM[Amide] | 1277.701 |
| Sensorin A | ARYRVGYMF[Amide] | 1161.606 |
| SCP | MNYLAFPRM[Amide] | 1141.572 |
| Sensorin A | TRSKNNVPRRFPRARYRVGYMF[Amide] | 2770.514 |
| apSPTR-DG-DP | MRYMGIC(-1.01)MKKQYNNFIPFPC(-1.01)LRS[Amide] | 2837.373 |
| AMRPs | SSEFPTSNSEQLALDT | 1725.787 |
| apUII | SGGMSLC(-1.01)LWKVC(-1.01)PAAPWLIS | 2117.024 |
| apUII | FKSPMQSRSGGMSLC(-1.01)LWKVC(-1.01)PAAPWLIS | 3078.504 |
| FRFa | SAAAILSPADDALLVRADEGEEEGVGGSYPGIPLSLPGTAD | 3980.967 |
| apUII | SMLLSKYMALMNRLQEQRQQEGDLSDLVGSPYVQQRSQ | 4427.196 |
| Lipid dimer | (C_42_H_85_NO_7_P)_2_ | 1492.213 |
| Lipid | C_42_H_85_NO_7_P | 746.606 |
| **Cerebral Ganglion** | | |
| Prohormone | Peptide | Monoisotopic *m/z* |
| apSPTR-DG-DP | MRYMGIC(-1.01)MKKQYNNFIPFPC(-1.01)LRS[Amide] | 2837.373 |
| Sensorin A | TRSKNNVPRRFPRARYRVGYMF[Amide] | 2770.514 |
| apSPTR-DG-DP | LPASRTDDVLQEASGLALN | 1970.025 |
| apUII | SGGMSLC(-1.01)LWKVC(-1.01)PAAPWLIS | 2117.024 |
| SCP | ARPGYLAFPRM[Amide] | 1277.701 |
| FMRGFamide | ELVESLGGED | 1047.490 |
| ELH | SSGVSLLTSNKDEEQRELLKAISNLLD | 2959.564 |
| Cerebrin | VRTYGNDLDARARREIISLAARLIKLSMYGPEDDSFV | 4211.209 |
| CP2 | FDFGFAGLDTYDAIHRALEQPARGTSNSGSGYNMLMKMQRH[Amide] | 4589.174 |
| Lipid dimer | (C_42_H_85_NO_7_P)_2_ | 1492.213 |
| Lipid | C_42_H_85_NO_7_P | 746.606 |
| **Pedal Ganglion** | | |
| Prohormone | Peptide | Monoisotopic *m/z* |
| PEP | PLDSVYGTHGMSGFA | 1538.700 |
| apSPTR-DG-DP | MRYMGIC(-1.01)MKKQYNNFIPFPC(-1.01)LRS[Amide] | 2837.373 |
| apSPTR-DG-DP | LPASRTDDVLQEASGLALN | 1970.025 |
| Myomodulin | GSYRMMRLGRGLHMLRL[Amide] | 2046.122 |
| AMRPs | SSEFPTSNSEQLALDT | 1725.787 |
| SCP | ARPGYLAFPRM[Amide] | 1277.701 |
| FCAP | ALDSLGGFQVHGW | 1386.686 |
| Buccalin | GLDRYGFVGGL[Amide] | 1152.624 |
| FMRGFamide | FLRGLNNYA | 1067.569 |
| NPY | DNSEMLAPPPRPEEFTSAQQLRQYLAALNEYYSIMGRPRF[Amide] | 4685.310 |
| Lipid dimer | (C_42_H_85_NO_7_P)_2_ | 1492.213 |
| Lipid | C_42_H_85_NO_7_P | 746.606 |
| **Pleural Ganglion** | | |
| Prohormone | Peptide | Monoisotopic *m/z* |
| SCP | ARPGYLAFPRM[Amide] | 1277.701 |
| Sensorin A | ARYRVGYMF[Amide] | 1161.606 |
| Sensorin A | TRSKNNVPRRFPRARYRVGYMF[Amide] | 2770.514 |
| apSPTR-DG-DP | MRYMGIC(-1.01)MKKQYNNFIPFPC(-1.01)LRS[Amide] | 2837.373 |
| MM2 | SLPDAGPADYPSLEDYLVQS | 2137.003 |
| FMRFa | SQEPDIEDYARAIALIESEEPLY | 2651.278 |
| apSPTR-DG-DP | LPASRTDDVLQEASGLALN | 1970.025 |
| AMRPs | SSEFPTSNSEQLALDT | 1725.787 |
| SCP | MNYLAFPRM[Amide] | 1141.572 |
| AMRPs | GEDVSERDYAQLLEALSRLQAIKQIKARIQNE | 3684.973 |
| Lipid dimer | (C_42_H_85_NO_7_P)_2_ | 1492.213 |
| Lipid | C_42_H_85_NO_7_P | 746.606 |

**Table S3.** Abundant, known peptides and lipids used to recalibrate the mass spectra from each ganglion.

| **Abdominal Ganglion** | | |
| --- | --- | --- |
| Prohormone | Peptide | Monoisotopic *m/z* |
| Lipid | C_42_H_85_NO_7_P | 746.606 |
| Sensorin A | ARYRVGYMF[Amide] | 1161.606 |
| R3-14 | [p-]EAEEPSAFMTRL | 1362.642 |
| R3-14 | EAEEPSAFMTRL | 1380.652 |
| Lipid dimer | (C_42_H_85_NO_7_P)_2_ | 1492.213 |
| AMRPs | SSEFPTSNSEQLALDT | 1725.787 |
| L11 | RPRIDC(-1.01)TRFVFAPAC(-1.01)RGVSA | 2220.149 |
| NPY | [p-]EFFRTNGERYPEDAAAWTEFQ | 2546.143 |
| FMRFa | SQEPDIEDYARAIALIESEEPLY | 2651.278 |
| Sensorin A | TRSKNNVPRRFPRARYRVGYMF[Amide] | 2770.514 |
| apSPTR-DG-DP | MRYMGIC(-1.01)MKKQYNNFIPFPC(-1.01)LRS[Amide] | 2837.373 |
| ELH | SSGVSLLTSNKDEEQRELLKAISNLLD | 2959.564 |
| AMRPs | GEDVSERDYAQLLEALSRLQAIKQIKARIQNE | 3684.973 |
| ELH | ISINQDLKAITDMLLTEQIRERQRYLADLRQRLLEK[Amide] | 4382.453 |
| NPY | DNSEMLAPPPRPEEFTSAQQLRQYLAALNEYYSIMGRPRF[Amide] | 4685.310 |
| **Bag Cell Clusters** | | |
| Prohormone | Peptide | Monoisotopic *m/z* |
| Lipid | C_42_H_85_NO_7_P | 746.606 |
| ELH | APRLRFYSL | 1122.632 |
| R3-14 | EAEEPSAFMTRL | 1380.652 |
| Lipid dimer | (C_42_H_85_NO_7_P)_2_ | 1492.213 |
| ELH | SVLTPSLSSLGESLESGIS | 1862.965 |
| ELH | TSNKDEEQRELLKAISNLLD | 2316.194 |
| ELH | SSGVSLLTSNKDEEQRELLKAISNLLD | 2959.564 |
| ELH | DQDEGNFRRFPTNAVSMSADENSPFDLSNEDGAVYQ | 4022.726 |
| ELH | ISINQDLKAITDMLLTEQIRERQRYLADLRQRLLEK[Amide] | 4382.453 |
| R3-14 | [p-]QVAQMHVWRAVNHDRNHGTGSGRHGRFLIRNRYRYGGGHLSDA | 4922.445 |
| **Buccal Ganglion** | | |
| Prohormone | Peptide | Monoisotopic *m/z* |
| Lipid | C_42_H_85_NO_7_P | 746.606 |
| SCP | MNYLAFPRM[Amide] | 1141.572 |
| Sensorin A | ARYRVGYMF[Amide] | 1161.606 |
| SCP | ARPGYLAFPRM[Amide] | 1277.701 |
| Lipid dimer | (C_42_H_85_NO_7_P)_2_ | 1492.213 |
| AMRPs | SSEFPTSNSEQLALDT | 1725.787 |
| apUII | SGGMSLC(-1.01)LWKVC(-1.01)PAAPWLIS | 2117.024 |
| FMRFa | SQEPDIEDYARAIALIESEEPLY | 2651.278 |
| Sensorin A | TRSKNNVPRRFPRARYRVGYMF[Amide] | 2770.514 |
| apSPTR-DG-DP | MRYMGIC(-1.01)MKKQYNNFIPFPC(-1.01)LRS[Amide] | 2837.373 |
| apUII | FKSPMQSRSGGMSLC(-1.01)LWKVC(-1.01)PAAPWLIS | 3078.504 |
| FRFa | SAAAILSPADDALLVRADEGEEEGVGGSYPGIPLSLPGTAD | 3980.967 |
| FRFa | SNLDLLRDVLSQYQLSAYPDAYPDAYPDAYIDDVDS | 4080.893 |
| apUII | SMLLSKYMALMNRLQEQRQQEGDLSDLVGSPYVQQRSQ | 4427.196 |
| **Cerebral Ganglion** | | |
| Prohormone | Peptide | Monoisotopic *m/z* |
| Lipid | C_42_H_85_NO_7_P | 746.606 |
| FMRGFamide | ELVESLGGED | 1047.490 |
| SCP | ARPGYLAFPRM[Amide] | 1277.701 |
| Lipid dimer | (C_42_H_85_NO_7_P)_2_ | 1492.213 |
| Insulin | DTENVNDKLRGILLN | 1713.919 |
| apSPTR-DG-DP | LPASRTDDVLQEASGLALN | 1970.025 |
| Sensorin A | TRSKNNVPRRFPRARYRVGYMF[Amide] | 2770.514 |
| apSPTR-DG-DP | MRYMGIC(-1.01)MKKQYNNFIPFPC(-1.01)LRS[Amide] | 2837.373 |
| ELH | SSGVSLLTSNKDEEQRELLKAISNLLD | 2959.564 |
| Cerebrin | VRTYGNDLDARARREIISLAARLIKLSMYGPEDDSFV | 4211.209 |
| ELH | ISINQDLKAITDMLLTEQIRERQRYLADLRQRLLEK[Amide] | 4382.453 |
| CP2 | FDFGFAGLDTYDAIHRALEQPARGTSNSGSGYNMLMKMQRH[Amide] | 4589.174 |
| **Pedal Ganglion** | | |
| Prohormone | Peptide | Monoisotopic *m/z* |
| Lipid | C_42_H_85_NO_7_P | 746.606 |
| Buccalin | GLDRYGFVGGL[Amide] | 1152.624 |
| SCP | ARPGYLAFPRM[Amide] | 1277.701 |
| FCAP | ALDSLGGFQVHGW | 1386.686 |
| Lipid dimer | (C_42_H_85_NO_7_P)_2_ | 1492.213 |
| PEP | PLDSVYGTHGMSGFA | 1538.700 |
| apSPTR-DG-DP | LPASRTDDVLQEASGLALN | 1970.025 |
| apSPTR-DG-DP | MRYMGIC(-1.01)MKKQYNNFIPFPC(-1.01)LRS[Amide] | 2837.373 |
| PEP | PLDSVYGTHGMSGFAKRPLDSVYGTHGMSGFA | 3342.573 |
| NPY | DNSEMLAPPPRPEEFTSAQQLRQYLAALNEYYSIMGRPRF[Amide] | 4685.310 |
| **Pleural Ganglion** | | |
| Prohormone | Peptide | Monoisotopic *m/z* |
| Lipid | C_42_H_85_NO_7_P | 746.606 |
| FMRGFamide | FLRGLNNYA | 1067.569 |
| SCP | MNYLAFPRM[Amide] | 1141.572 |
| Sensorin A | ARYRVGYMF[Amide] | 1161.606 |
| SCP | ARPGYLAFPRM[Amide] | 1277.701 |
| FMRFa | SMSVEEPHFRLE | 1460.690 |
| Lipid dimer | (C_42_H_85_NO_7_P)_2_ | 1492.213 |
| AMRPs | SSEFPTSNSEQLALDT | 1725.787 |
| apSPTR-DG-DP | LPASRTDDVLQEASGLALN | 1970.025 |
| MM2 | SLPDAGPADYPSLEDYLVQS | 2137.003 |
| FMRFa | SQEPDIEDYARAIALIESEEPLY | 2651.278 |
| Sensorin A | TRSKNNVPRRFPRARYRVGYMF[Amide] | 2770.514 |
| apSPTR-DG-DP | MRYMGIC(-1.01)MKKQYNNFIPFPC(-1.01)LRS[Amide] | 2837.373 |
| AMRPs | GEDVSERDYAQLLEALSRLQAIKQIKARIQNE | 3684.973 |
